# Optimized Peptide–MHC Multimer Protocols for Detection and Isolation of Autoimmune T-Cells

**DOI:** 10.3389/fimmu.2018.01378

**Published:** 2018-06-29

**Authors:** Garry Dolton, Efthalia Zervoudi, Cristina Rius, Aaron Wall, Hannah L. Thomas, Anna Fuller, Lorraine Yeo, Mateusz Legut, Sophie Wheeler, Meriem Attaf, Dmitriy M. Chudakov, Ernest Choy, Mark Peakman, Andrew K. Sewell

**Affiliations:** ^1^Division of Infection and Immunity, Cardiff University School of Medicine, Cardiff, United Kingdom; ^2^Department of Immunobiology, Faculty of Life Sciences and Medicine, King’s College London, London, United Kingdom; ^3^NIHR Biomedical Research Centre at Guy’s and St Thomas’ NHS Foundation Trust and King’s College London, London, United Kingdom; ^4^Pirogov Russian National Research Medical University, Moscow, Russia; ^5^Centre for Data-Intensive Biomedicine and Biotechnology, Skolkovo Institute of Science and Technology, Skolkovo, Russia; ^6^Shemyakin and Ovchinnikov Institute of Bioorganic Chemistry, Moscow, Russia; ^7^Systems Immunity Research Institute, Cardiff University, Cardiff, United Kingdom

**Keywords:** peptide–MHC multimer, tetramer, dextramer, autoimmune disease, T-cell, type 1 diabetes, ankylosing spondylitis, cancer epitope

## Abstract

Peptide–MHC (pMHC) multimers have become the “gold standard” for the detection and isolation of antigen-specific T-cells but recent evidence shows that normal use of these reagents can miss fully functional T-cells that bear T-cell receptors (TCRs) with low affinity for cognate antigen. This issue is particularly pronounced for anticancer and autoimmune T-cells as self-reactive T-cell populations are enriched for low-affinity TCRs due to the removal of cells with higher affinity receptors by immune tolerance mechanisms. Here, we stained a wide variety of self-reactive human T-cells using regular pMHC staining and an optimized technique that included: (i) protein kinase inhibitor (PKI), to prevent TCR triggering and internalization, and (ii) anti-fluorochrome antibody, to reduce reagent dissociation during washing steps. Lymphocytes derived from the peripheral blood of type 1 diabetes patients were stained with pMHC multimers made with epitopes from preproinsulin (PPI), insulin-β chain, glutamic acid decarboxylase 65 (GAD65), or glucose-6-phospate catalytic subunit-related protein (IGRP) presented by disease-risk allelles HLA A*02:01 or HLA*24:02. Samples from ankylosing spondylitis patients were stained with a multimerized epitope from vasoactive intestinal polypeptide receptor 1 (VIPR1) presented by HLA B*27:05. Optimized procedures stained an average of 40.5-fold (*p* = 0.01, range between 1.4 and 198) more cells than could be detected without the inclusion of PKI and cross-linking anti-fluorochrome antibody. Higher order pMHC dextramers recovered more cells than pMHC tetramers in parallel assays, and standard staining protocols with pMHC tetramers routinely recovered less cells than functional assays. HLA A*02:01-restricted PPI-specific and HLA B*27:05-restricted VIPR1-specific T-cell clones generated using the optimized procedure could not be stained by standard pMHC tetramer staining. However, these clones responded well to exogenously supplied peptide and endogenously processed and presented epitopes. We also showed that anti-fluorochrome antibody-conjugated magnetic beads enhanced staining of self-reactive T-cells that could not be stained using standard protocols, thus enabling rapid *ex vivo* isolation of autoimmune T-cells. We, therefore, conclude that regular pMHC tetramer staining is generally unsuitable for recovering self-reactive T-cells from clinical samples and recommend the use of the optimized protocols described herein.

## Introduction

**Figure 1:**
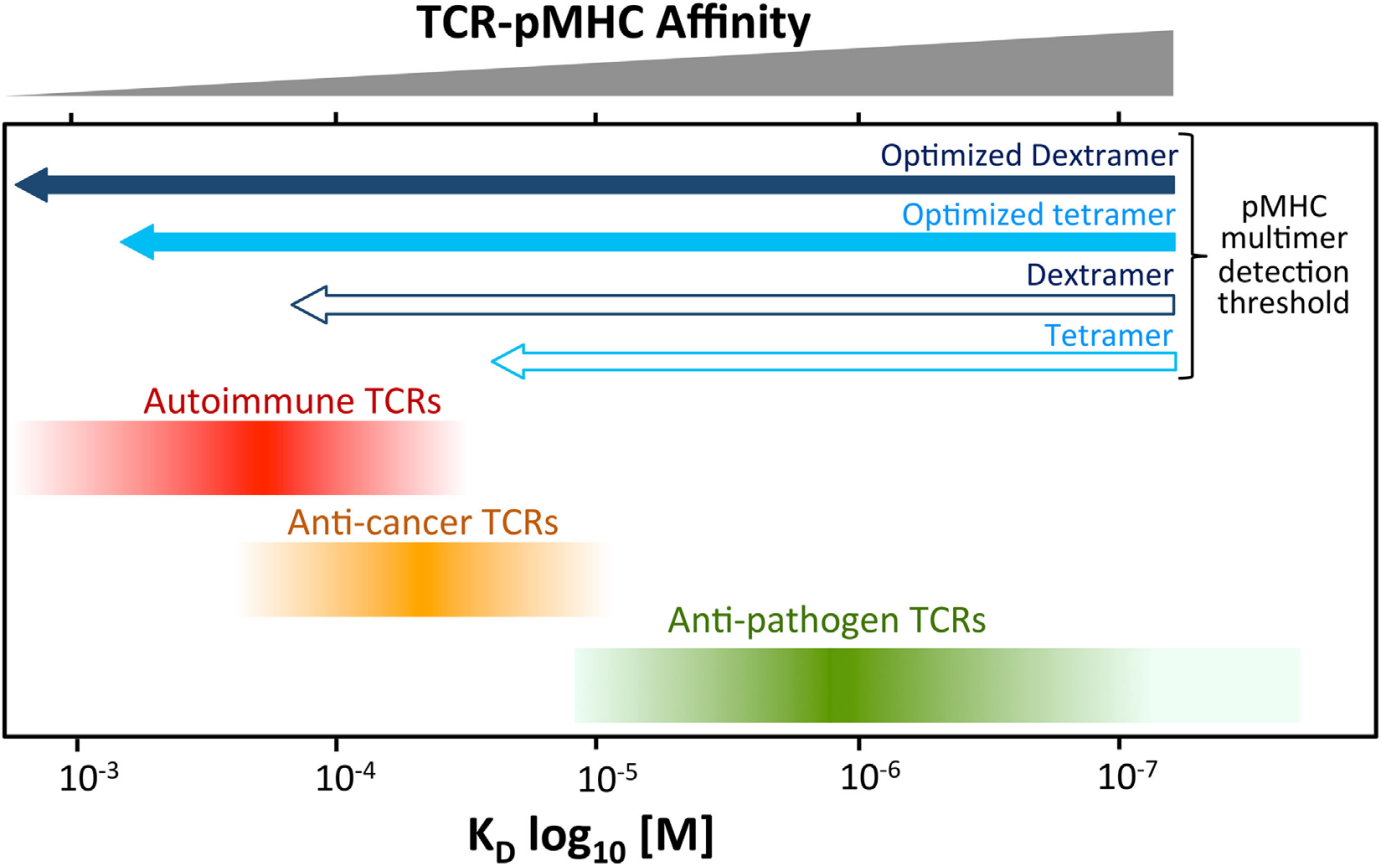
Self-reactive T-cells have low-affinity T-cell receptors (TCRs). Anti-pathogen TCRs tend to bind to cognate Peptide–MHC (pMHC) with relatively high affinity (*K*_D_ < 20 μM). The rigors of central tolerance ensure that autoimmune TCRs bind with much weaker affinity. Anticancer TCRs tend to sit between these two extremes. This schematic depicts a general overview of what is normally observed. A minority of TCRs do not obey these general rules. Cancer TCRs that bind to unstable pMHC have been described can have higher affinites (*K*_D_ ~ 15 µM) (16). TCRs specific for cancer neoantigens (non-self) may behave more like pathogen-specific TCR. The arrows at the top indicate the rough affinity detection threshold of TCRs amenable to staining with standard pMHC multimer staining and an optimized procedure including protein kinase inhibitor and antibody cross-linking. Optimized staining with pMHC tetramers is believed to detect almost all antigen-specific T-cells. An optimized dextramer is only required when staining the very weakest of functional autoimmune T-cells.

Conventional T-cells orchestrate the immune response to pathogens by recognizing foreign protein antigens in the form of peptides presented at the cell surface bound to MHC molecules. The key to recognition lies in the heterodimeric αβ T-cell receptor (TCR) which, in concert with either the CD4 or CD8 coreceptor, engages peptide–MHC (pMHC) to produce an intracellular transduction cascade that results in T-cell activation (1–3). TCR–pMHC binding parameters are selected in the thymus *via* recruitment of the signal-initiating kinase Lck, which is sequestered by the intracellular tails of CD4 and CD8 (4). These coreceptors bind to sites on MHC class I and class II, respectively, that are distinct from the TCR-docking platform thereby enabling formation of TCR–pMHCI-CD8 or TCR–pMHCII-CD4 quadripartite complexes (3, 4). The privileged delivery of Lck to the cytoplasmic side of the TCR/CD3 complex by the T-cell coreceptors ensures that TCRs are MHC-restricted (5–7) and selects the TCR–pMHC dwell time that permits onward thymic development and release into the periphery (4). T-cells bearing TCRs that do not interact with self pMHC die by neglect in the absence of a positive selection signal (8). At the other extreme, cells bearing TCRs that bind strongly to self pMHC, and thereby have potential to react to self, are eliminated from the pool of developing T-cells (8). This central tolerance mechanism ensures that T-cells with TCRs that interact strongly with self-peptides do not enter the peripheral tissues and, in conjunction with peripheral tolerance mechanisms, explains why self-reactive TCRs bind with lower affinity, and with shorter dwell times than TCRs specific for foreign, pathogen-derived peptides (9, 10). Indeed, the best anti-pathogen TCRs tend to bind with TCR affinities with dissociation constants (*K*_D_s) in the range of 0.1–10 µM by surface plasmon resonance (SPR) whereas self-reactive TCRs isolated from anticancer and autoimmune T-cells bind with much weaker affinities (*K*_D_ 20–500 µM) (9–11). Functional assays using modified TCRs with enhanced affinities indicate that the most sensitive T-cells tend to have TCRs that bind with K_D_s between 0.1 and 3 µM, which sit at the higher end of the natural anti-pathogen TCR affinity range (9–11). Further engineered increases in TCR affinity are generally detrimental as they are thought to impede the serial triggering of many TCRs by a single agonist pMHC and can result in recognition of self-peptides (12, 13). Thus, T-cell development imposes a dwell time “window” on the TCRs of peripheral T-cells that is of sufficient duration to allow TCR triggering but short enough to enable serial triggering of multiple receptors by each agonist pMHC (14). In general, anti-pathogen TCRs sit at the stronger end of this affinity window, followed by anticancer TCRs (1, 9, 10), whereas autoimmune TCRs reside at the other end of the spectrum and can have TCRs that bind very weakly (*K*_D_ > 150 μM) [(10, 15) and unpublished data] (Figure 1).

Although TCR engagement with cognate pMHC is short-lived, this interaction can be stabilized by the “avidity effect” afforded by incorporating multiple pMHC on a single molecule (17). This effect was originally taken advantage of by combining soluble biotinylated pMHC with fluorochrome-conjugated streptavidin. The resultant pMHC tetramers can bind stably to cognate T-cells within peripheral blood mononuclear cells (PBMC) to allow detection and phenotyping of antigen-specific T-cells directly *ex vivo*. Since their initial adoption in 1996 (18), pMHC tetramers have been used in many thousands of studies and it is no exaggeration to say that they have revolutionized the study of antigen-specific T-cell populations in *ex vivo* samples (19). Various pMHC multimerization platforms have been utilized, most of which are commercially available [reviewed in Ref. (17, 20)]. In 2007, we described how the affinity required for stabilization of pMHC tetramer binding was considerably higher than that required for T-cell activation (21). Consequently, standard pMHC tetramer staining fails to reveal fully functional T-cells that bear TCRs below the limits of detection. This deficiency precipitated the development of various methodologies aimed at lowering the TCR affinity threshold for pMHC multimer staining (summarized in Table 1).

**Table 1:** Methods for improving peptide–MHC (pMHC) multimer staining.

Method	Mechanism of action	Pros	Cons	Reference
Higher order multimers	Increased avidity due to more TCR interactions per molecule	Detects T-cells with l ower affinity TCRs. Can carry more fluorochome resulting in brighter staining	More expensive and more complex for DIY construction	(22, 23)
Enhancing coreceptor Ab	Enhances rather than inhibits TCR–pMHC interactions	Works for MHCI and MHCII	None known	(24–27)
Enhanced MHC–CD8 interaction	Mutation of the MHC at the CD8 binding site that stabilizes TCR–pMHCI interactions	Allows stable interaction with lower affinity TCRs	Only applicable to MHCI and can increase non-specific background	(17, 28, 29)
Altered peptide flanking regions	Enhances TCR–pMHCII interactions	Can substantially enhance staining of CD4 T-cells with multimerized MHCII	Only applicable to MHCII. May differentially favor TCRs with certain TRBV genes	(30, 31)
Protein kinase inhibitor	Prevents TCR triggering and downregulation	Very inexpensive. Applicable to any pMHC multimer in all species tested to date	None known	(32)
Cross-linking Ab	Stabilizes bound pMHC multimer during washing steps	Very inexpensive. Applicable to any pMHC multimer in all species tested to date	None known	(33)

*Only the methods boxed in red were utilized in this study*.

We currently utilize an optimized pMHC multimer staining procedure that incorporates two simple, inexpensive, broadly applicable improvements that can be used with pMHCI and pMHCII reagents and aids staining in all species tested to date, including human, mouse, monkey, and pig (32–35). The first of these improvements involves inclusion of 50nM Dasatinib, a protein kinase inhibitor (PKI), to prevent TCR downregulation (32). In addition, we include an anti-fluorochrome antibody (Ab) to cross-link pMHC multimers at the T-cell surface. Such cross-linking can result in substantial improvements in pMHC multimer staining by stabilizing binding and limiting dissociation during washing steps (33). Here, we apply standard pMHC tetramer staining and an optimized protocol including PKI and cross-linking Ab (PKI + Ab) when staining clinical samples with a wide range of self-epitopes relevant to type 1 diabetes (T1D) and ankylosing spondylitis. The optimized protocol was found to be up to two orders of magnitude more effective, in terms of cell numbers recovered and brightness of staining, than parallel staining in the absence of PKI + Ab. Importantly, T-cell clones isolated by optimized pMHC multimer staining could not be stained with cognate antigen in the absence of PKI + Ab despite being fully functional, i.e., able to recognize targets in the presence of exogenously supplied peptide or endogenously processed and presented antigen.

## Materials and Methods

### Patient Material and Ethical Approval Statement

Cryopreserved PBMCs from patients with T1D were obtained with the approval of a national research ethics committee, and informed consent was obtained from all participants. Ankylosing spondylitis patients were recruited locally from the clinic (Cardiff Regional Experimental Arthritis Treatment and Evaluation Centre) and anonymized whole blood used for preparation of PBMCs. Patients gave informed consent in accordance with the Research Ethics Committee for Wales (12/WA/0045). PBMC were obtained from a further HLA B*27:05 ankylosing spondylitis patient diagnosed at the Institute of Rheumatology, Russian Academy of Medical Sciences, Moscow, Russia, in accordance with modified New York criteria (36). This patient gave written informed consent prior to enrolling in the study. The study was approved by the local ethical committee of Pirogov Russian National Research Medical University, Moscow, Russia. This patient has been the subject of several previous studies (37–39).

### Peptide Epitopes

The peptide epitopes and their restricting HLA and disease relevance are listed in Table 2.

**Table 2:** T-cell epitopes used in this study.

HLA-restriction	Peptide sequence	Protein origin (and residues)	Disease relevance	Reference
A*02:01	NLVPMVATV	Cytomeglavirus (CMV) pp65 (495–503)	CMV infection (control)	(40)
A*02:01	ILAKFLHWL	Human telomerase reverse transcriptase (hTERT) (540–548)	Not natural (control)	(41)
A*02:01	ALWGPDPAAA	Preproinsulin (PPI) (15–24)	Type 1 diabetes (T1D)	(42)
A*02:01	VMNILLQYVV	Glutamate decarboxylase 65 (GAD65) (114–123)	T1D	(43)
A*02:01	VLFGLGFAI	Islet-specific glucose-6-phosphatase catalytic subunit-related protein (IGRP) (265–273)	T1D	(44)
A*02:01	MVWESGCTV	Islet Antigen-2 (IA-2) (795–805)	T1D	(45)
A*02:01	HLVEALYLV	Insulin-β chain (10–18)	T1D	(46)
A*02:01	NLSALGIFST	Insulin-like growth factor 2 mRNA binding protein 2 (IMP2) (367–376)	Cancer	Unpublished
A*24:02	LWMRLLPLL	PPI (3–11)	T1D	(47)
B*27:05	KRWILLGLNK	HIV p24 gag (263–27)	HIV infection (control)	(48)
B*27:05	RRKWRRWHL	Vasoactive intestinal polypeptide receptor 1 (VIPR1) (400–408)	Ankylosing spondylitis	(49)
B*27:05	DRASFIKNL	Collagen type VI α2 subunit (114–122)	Ankylosing spondylitis	(50)

### T-Cell Culture and Cloning

T-cell clones and lines were cultured in T-cell media (RPMI-1640, 10% fetal bovine serum (FBS), 2 mM l-glutamine, 100 U/mL penicillin, 100 µg/mL streptomycin, 10 mM HEPES buffer, 0.5× non-essential amino acids, and 1 mM sodium pyruvate, all from Life Technologies, Carlsband, CA, USA) supplemented with 200 IU of IL-2 (Aldesleukin, Proleukin, Prometheus, San Diego, CA, USA) and 25 ng/mL human IL-15 (Peprotech, Rocky Hill, NJ, USA). T-cells were stimulated for expansion every 2–5 weeks with irradiated (3,000–3,100 cGy) allogeneic PBMCs from three donors and 1–2 µg/mL of phytohemagglutinin (Alere, Thermo Scientific, Walthan, MA, USA). T-cell clones were procured by limiting dilution as previously described (51) and expanded as described above. T-cells were cultured for a minimum of 2 weeks post-restimulation before being used for experiments.

### Generation of Immortalized B-Cells

Lymphoblastoid cell lines (LCLs) were created from PBMCs by incubation with Epstein–Barr virus (EBV) containing supernatant from the B95-8 cell line. Media (36–48 h of culture) from Cotton-top Tamarin monkey B95-8 cell line (European Collection of Authenticated Cell Cultures, catalogue number 85011419) at 80–90% confluence was harvested, centrifuged at 400 *g* for 5 min, 0.22-µm filtered, and stored at -80°C. PBMCs (1–5 × 10^6^) were cultured in 1 mL R10 medium (RPMI, 10% FCS, l-glutamine, penicillin, and streptomycin) with 1 mL of B95-8 supernatant with 4 µg/mL of cyclosporin A (Sigma-Aldrich, St. Louis, MO, USA). Cells were passaged as required, and cyclosporin A treatment continued for 2 weeks.

### Expressing Transgenic Self-Proteins

A K562 cell line expressing HLA A*02:01 and preproinsulin was generated and cultured as previously described (42). Patient LCLs were made to express vasoactive intestinal polypeptide receptor 1 (VIPR1) or the α2 chain of collagen type VI. Codon optimized full-length *VIPR1* (UniProtKB P32241) or *COL6A2* (UniProtKB P12110) cDNAs were synthesized (Genewiz, South Plainfield, NJ, USA) and cloned into the third generation lentiviral transfer vector pELNS (kindly provided by Dr. James Riley, University of Pennsylvania, PA, USA). The pELNS vector contains a rat CD2 (rCD2) marker gene separated from the gene of interest by a self-cleaving 2A sequence. Lentiviral particle production by calcium chloride transfection and rCD2-based purification of lentivirally transduced cells were performed as previously described (52).

### pMHC Multimers and Flow Cytometry

Monomeric pMHCs were generated in-house (53) and used to assemble tetramer and dextramers as previously described (22, 33). Premium grade R-Phycoerythrin conjugated streptavidin (SA) was purchased from Life Technologies (catalog number S21388). R-Phycoerythrin conjugated SA dextramer backbone was sourced from Immudex Limited (Copenhagen, Denmark). Tetramers and dextramer were stored at 4°C in the dark with added protease inhibitors (1:100 of set 1, Merck, London, UK) and used within three days of being assembled. Clones/lines/PBMCs were harvested from culture or recently defrosted cells, washed (400 *g*, 5 min), counted, and placed in 5mL polypropylene tubes suitable for flow cytometry. Typically, 2 × 10^4^ of a clone, 1 × 10^5^ of a line, or 3 × 10^6^ PBMCs were used for staining. The cells were washed with FACS buffer (PBS + 2% FBS).

#### Optimized pMHC Multimer Staining

An overview of the optimized staining protocols used in this study can be found in Figure S1 in Supplementary Material. Cells were pretreated with the PKI (50 nM) Dasatinib (Axon Medchem, VA, USA), for 5–60 min (typically 30 min) at 37°C in 100 µL:50 µL of residual fluid and 50 µL of 2× PKI (100 nM) in PBS. PKI was stored as 1 mM single use DMSO stocks at -80°C. The flexibility of the incubation time needed for PKI allows either a “fast-track” staining protocol (5 min with PKI) to be performed or allows time to prepare other reagents needed for the assay (<60 min with PKI). PE-conjugated tetramers or dextramers were spun in a microfuge to remove aggregates (full speed for 1 min), and then 0.5 µg (with respect to pMHC component) added directly to each sample without washing or pre-chilling, followed by incubation for 30 min on ice and in the dark. Cells were washed with 3 mL of FACS buffer (700 g, 3 min) and 0.5 µg (10 µg/mL) of mouse anti-PE unconjugated Ab (clone PE001, BioLegend, London, UK) added to each sample for 20 min on ice and in the dark. Whereas we used PE-conjugated pMHC multimers for this study, other fluorochrome, for instance allophycocyanin (APC), conjugated tetramers or dextramers can also be used with the optimized protocol, by matching the anti-fluorochrome Ab to the pMHC multimer, as previously described (53). Cells were washed with 3 mL of PBS (700 *g*, 3 min) to remove residual serum and incubated for 5 min at RT with 2 µL of LIVE/DEAD fixable dead cell stain (Vivid; Life Technologies) that had been diluted 1:40 using PBS. Ab cocktails to detect surface markers were added directly to each sample without washing. For clones, anti-CD8-APC or -APC Vio770™ (clone BW135/80, Miltenyi Biotech) and anti-CD3-peridinin chlorophyll (clone BW264/56, Miltenyi Biotech) were used. For lines and PBMCs, anti-CD19-pacific blue (PB) (clone HIB19, BioLegend) and anti-CD14-PB (clone M5E2, BioLegend) Abs were also included. Cells were washed with 3 mL of FACS buffer and resuspended in 50 µL of FACS buffer. Although not performed in this study, samples can be fixed with 2% paraformaldehyde (PFA) by washing with 3 mL of PBS, incubating with 50 µL of 4% PFA (added to the 50 µL of residual PBS left after washing) on ice for 20 min in the dark, washing again with 3 mL of PBS, decanting, then storing at 4°C in the dark ready for flow cytometry the following day. Samples were vortexed throughout the staining protocol, both prior and post the addition of reagents.

#### Standard pMHC Staining

The standard protocol was performed in the same manner as the optimized, but without addition of PKI or anti-PE Ab. For the comparative purpose of this study, cells were mock treated with PKI by the addition of 50 µL of PBS.

#### Flow Cytometry and Analysis

For acquisition cells were run a BD FACS Canto II (BD Biosciences) and for sorting on a BD FACS Aria (BD Biosciences) run by Central Biotechnology Services (Cardiff University, Wales, UK). Sequential gating strategy for tetramer and dextramer analyses; Gate 1: lymphocytes; Gate 2: single cells; Gate 3: CD3^+^ Vivid^neg^ CD19^neg^ CD14^neg^ cells; then displayed as dot plots (CD8 versus tetramer or dextramer) or histograms of tetramer or dextramer fluorescence. Smoothed zebra plots with outliers shown as large dots were used for the display of dot plots. FlowJo software (TreeStar, Inc., Ashland, OR, USA) was used to analyze the data.

### Magnetic Bead Enrichment

Peripheral blood mononuclear cells were treated and incubated with PKI and dextramers as above (Figure S1 in Supplementary Material for protocol schematic). Cells were washed in ice cold MACS buffer (D-PBS without calcium and magnesium ions, 0.5% bovine serum albumin (both Sigma-Aldrich), and 2 mM EDTA, pH 7.2–7.5). Anti-PE magnetic microbeads were used according to the manufacturer’s instructions, whereby 80 µL of MACS buffer and 20 µL of beads were used per 1 × 10^7^ cells, with no scaling down for lower cell numbers. Positive cells were collected by centrifugation (400 *g* for 5 min) and incubated overnight in a single well of a 96-U-well plate, in T-cell media. Cells were expanded in the same well with PHA and allogenic PBMCs, as described above.

### Functional Assays

#### TNF Processing Inhibitor-0 (TAPI-0) Assay

Cells were harvested from culture washed with R0 (as for R10 but with no FBS) and rested overnight in R5 media (as for R10 but with 5% FBS). Resting ensured minimal background activation of the T-cells. On the day of activation assay, cells were harvested, and 2–5 × 10^4^ cells were incubated with 30 µM TAPI-0 (Sigma-Aldrich) (54) anti-TNF-PE-Vio770™ (clone cA2, Miltenyi Biotech) and anti-CD107a-PE (clone H4A3, BD Biosciences) Abs in well(s) of a 96U-well plate. For peptide presentation, autologous LCLs were used for clonal T-cells, and T-cell to T-cell presentation used for T-cell lines. The cells were incubated for 4–5 h at 37°C then stained with Vivid and Abs for CD3 and/or CD8, as above.

#### Enzyme-Linked Immunosorbent Assay (ELISA)

Prior to assay, T-cell clones were rested overnight in R5. Clonal CD8 T-cells (3 × 10^4^ per well) were incubated overnight at 37°C. Autologous LCLs or T2 cells (ATCC CRL-1992) were used as antigen presenting cells for clonal T-cells. Supernatants were harvested the following morning and assayed for macrophage inflammatory protein (MIP)-1β by ELISA according to the manufacturer’s instructions (R&D Systems, Minneapolis, MN, USA).

## Results

### Superior Detection of Pancreatic Peptide-Specific T-Cells With an Optimal Tetramer Staining Protocol

**Figure 2:**
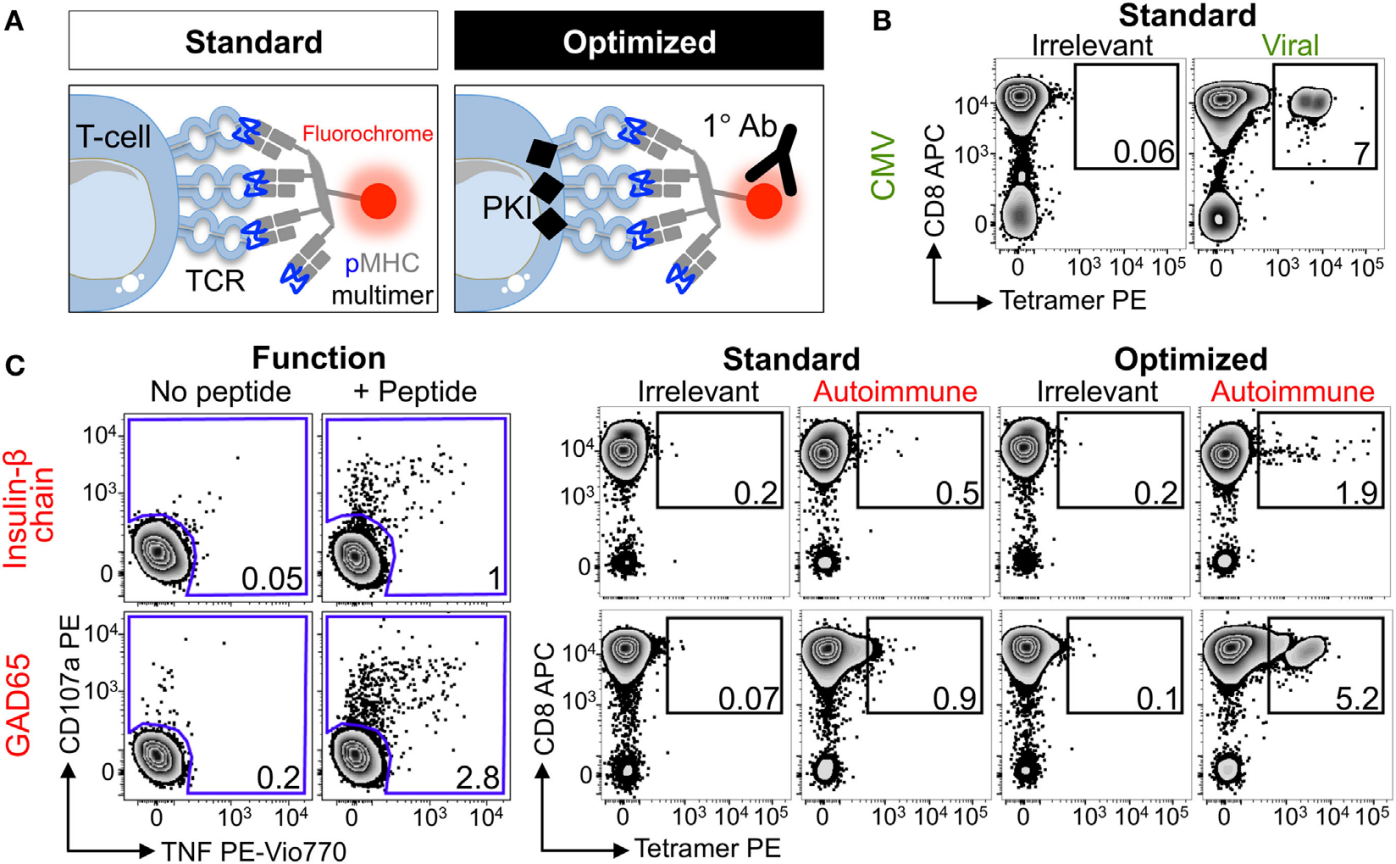
Enhanced detection of autoimmune T-cells from a type I diabetic patient using optimal tetramer staining techniques. **(A)** Standard and optimal tetramer staining approaches to detect antigen-specific T-cells. Optimal staining uses the protein kinase inhibitor (PKI) Dasatinib to treat T-cells before staining with tetramer and then an unconjugated anti-fluorochrome Ab. We have previously described the use of conjugated secondary antibodies to bind the primary antibody, adding further fluorescence to peptide–MHC multimers labeled T-cells, but for the purpose of this study only the primary cross-linking antibody was used. T-cell receptor (TCR). **(B)** Sorted CD8 T-cells from a HLA A*02:01^+^ patient with type I diabetes were stimulated with a peptide from CMV (pp65_495–503_, NLVPMVATV) and then stained 2 weeks later with irrelevant (hTERT_540–548_, ILAKFLHWL) or CMV tetramers using standard conditions. **(C)** From the same patient in **(B)** CD8 T-cells were stimulated with peptide (HLVEALYLV) from insulin-β chain_10–18_ or glutamate decarboxylase 65 (GAD65_114–123_, VMNILLQYVV). 2 weeks post-stimulation reactivity toward the respective peptide (100 nM) was assessed by co-incubation with TAPI-0 and detection of CD107a and TNF (left panel). The T-cell lines were stained with PE-conjugated irrelevant (as above) and respective autoimmune tetramers using standard or optimal conditions (right panel). The percentage of cells residing in each gate are shown.

Four HLA class I alleles have been linked to T1D, namely HLAs A*02:01, A*24:02, B*18:01, and B*39:06 (55). Due to the prevalence of some of these alleles in the general population, the vast majority of T1D patients carry either HLA A*02:01 or HLA A*24:02 so these alleles were chosen for this study. Several pancreatic β-cell-specific epitopes that are presented by these alleles have been defined (42, 44, 47, 56, 57). Purified CD8 T-cells from an HLA A*02:01^+^ type I diabetes patient were stimulated with peptides from CMV, GAD65, IGRP, IA-2, and the insulin-β chain (Table 2) to create T-cell lines for testing with standard and optimized tetramer staining protocols (Figure 2A) (see Figure S1 in Supplementary Material for optimized protocol schematic). The control CMV line stained well with the pp65 tetramer under standard conditions with clear separation between the tetramer^neg^ and tetramer^+^ cells (Figure 2B), which is characteristic of standard staining for many antiviral T-cell populations (22). Prior to cognate multimer staining, T-cell lines that had been stimulated with pancreatic β-cell-specific epitopes were assessed for reactivity against exogenously supplied peptides by measuring CD107a (58) and TNF (54) expression. No specific response was observed in lines stimulated with IGRP and IA2 peptides, and the lines were discarded. In contrast, the lines stimulated with insulin-β chain and GAD65 peptides expressed TNF/CD107a in response to cognate antigens (Figure 2C). These peptide-reactive T-cell lines were subsequently stained with irrelevant and cognate pancreatic peptide-HLA A*02:01 PE-conjugated tetramers. The percentage of tetramer^+^ cells increased when using the optimized protocol compared to the standard approach; in both cases, the optimized protocol resulted in approximately sixfold increase in the percentage of detected cells (Figure 2C). Importantly, the tetramer^+^ populations became more discernible from the tetramer^neg^ cells when using the optimized protocol. Furthermore, the percentage of peptide-specific cells detected with the optimized protocol correlated better with the functional response to exogenously supplied peptides, while standard tetramer staining vastly underestimated the fraction of peptide-reactive cells. Overall, the inclusion of two inexpensive reagents, the PKI Dasatinib and anti-fluorochrome Ab, to create an optimal tetramer staining protocol allowed autoimmune T-cells to be detected with relative ease.

### Optimal Staining With pMHC Dextramers Further Improves the Detection of Autoimmune T-Cells

**Figure 3:**
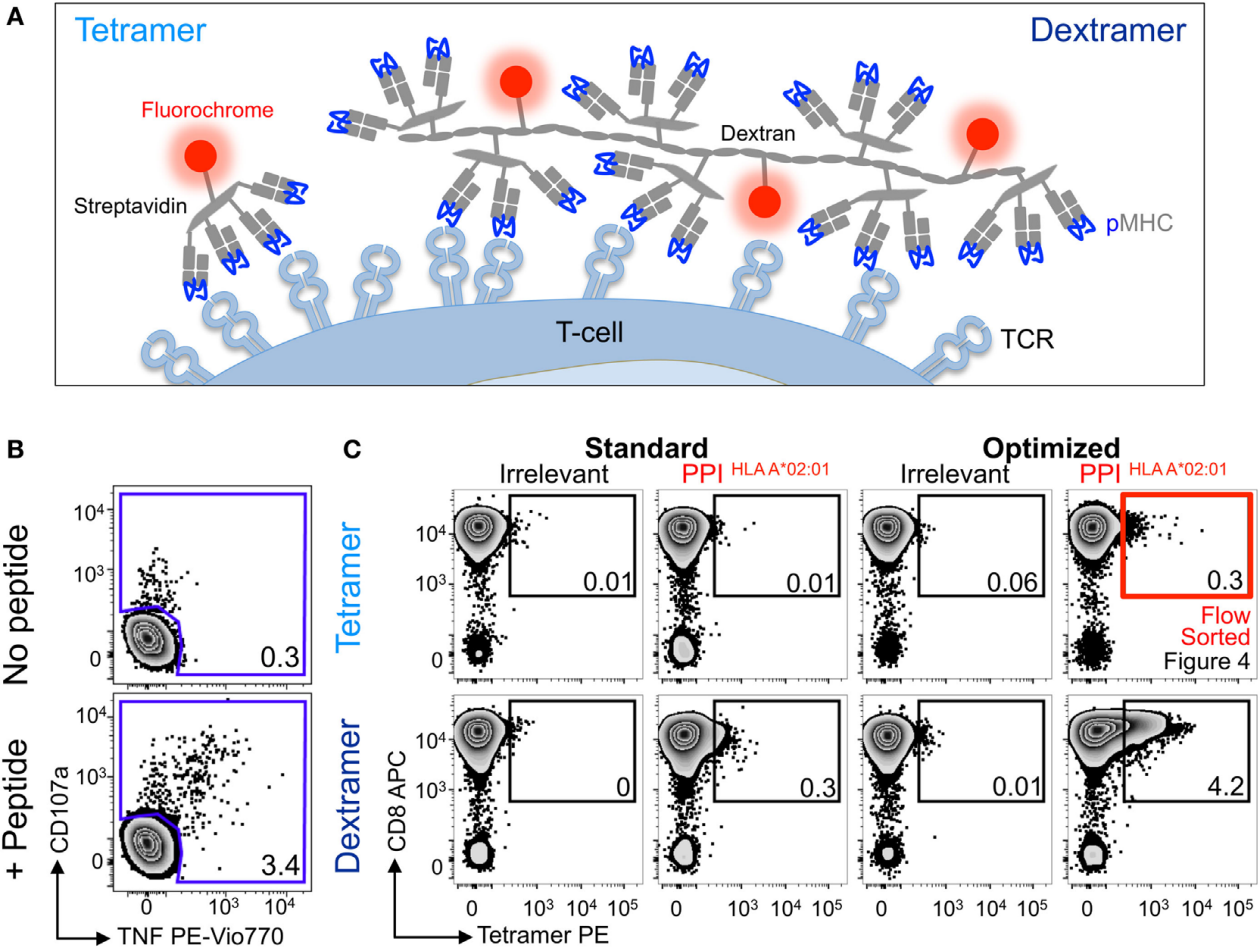
Detection of preproinsulin (PPI) T-cells is further improved using dextramers under optimal staining conditions. **(A)** Schematic representation of tetramers and dextramers showing the number of streptavidins (typically, 6–7 for dextramers), peptide–MHC (pMHCs), and phycoerythrins per unit of reagent. pMHC to streptavidin molar ratios of 4:1 and 3:1 were used to assemble tetramers and dextramers, respectively. T-cell receptor (TCR). **(B)** Sorted CD8 T-cells from a HLA A*02:01^+^ patient with type I diabetes were stimulated with a peptide from PPI (PPI_15–24_) and assessed 2 weeks later for reactivity against the PPI peptide (100 nM) by co-incubation with TAPI-0 and detection of CD107a and TNF. **(C)** The T-cells were also stained with irrelevant (hTERT_540–548_, ILAKFLHWL) or PPI tetramers (upper panel) and dextramers (lower panel) using standard (pMHC multimer alone) and optimal protocols (PKI + anti-PE Ab). The percentage of cells residing in each gate are shown. The red box indicates cells that were sorted by flow cytometry and shown in Figure 4.

We next examined the PPI_15–24_ T-cell line grown as above. Even using the optimized tetramer staining protocol, we managed to detect only 10-fold less cells than detected in the parallel functional assay (Figure 3A). We then turned to using pMHC dextramers as we had previously demonstrated that these higher order reagents can extend the TCR affinity threshold that is amenable to pMHC multimer staining (Figure 1 and reagent schematic in Figure 3A) (22). Standard pMHC dextramer staining recovered a population of CD8 T-cells of similar size to that recovered with an optimized pMHC tetramer procedure (Figure 3B). Use of the optimized procedure in conjunction with pMHC dextramer increased the size of the detected population by over 10-fold and brought it into line with the population size observed in the functional assay. While optimized dextramer staining vastly outperformed optimized tetramer staining in this case, the cells detected using the latter were fully functional. To formally prove functionality, we sorted cells that stained with optimized tetramer protocol and used them for further analysis (Figure 3C).

### Optimally Tetramer-Sorted PPI-Specific T-Cell Clones Do Not Stain With a Standard pMHC Tetramer Protocol

**Figure 4:**
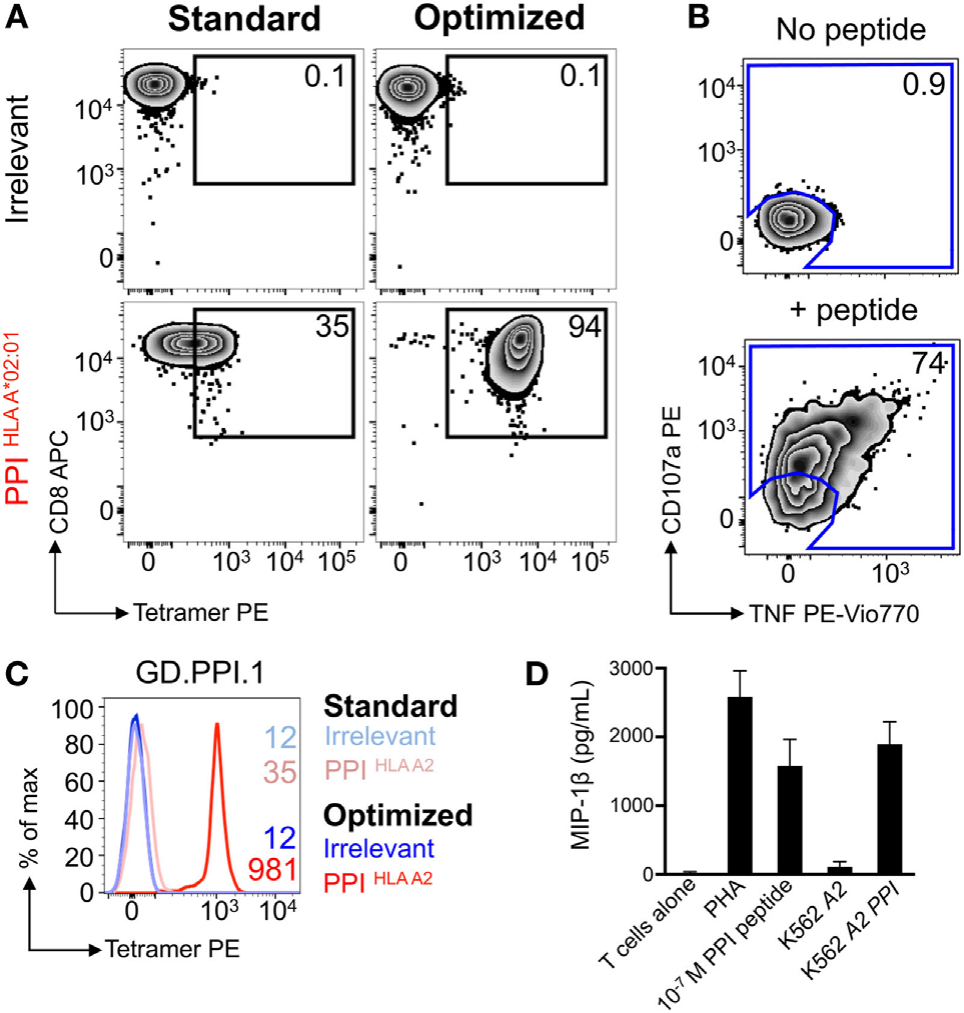
Optimal staining with preproinsulin (PPI) tetramers allowed functional T-cells to be isolated. Peripheral blood mononuclear cells (PBMCs) from a HLA A*02:01^+^ patient with type I diabetes were stimulated with PPI (PPI_15–24_) peptide (Figure 3A), which stained with PPI tetramer and dextramer under optimal conditions (PKI + anti-PE Ab). Tetramer^+^ cells were sorted by flow cytometry (Figure 3B) and expanded with PHA and irradiated allogeneic PBMCs. **(A)** The expanded cells were stained with irrelevant (ILAKFLHWL, hTERT_540–548_) and PPI tetramers using standard (tetramer alone) and optimal (as above) protocols. The percentage of cells residing in each gate is shown. **(B)** Reactivity of the enriched T-cells for PPI peptide was assessed by co-incubation with TAPI-0 and detection of CD107a and TNF. **(C)** Clone GD.PPI.1 grown from the enriched line was stained with irrelevant (hTERT) and PPI tetramers using standard and optimal protocols. The MFI of staining is shown on the histogram according to the key. **(D)** Overnight activation assay and MIP-1β enzyme-linked immunosorbent assay, with PHA as a positive control, PPI peptide (10 nM), and K562s transduced with genes for HLA A*02:01 (*A2*) ± PPI cDNA (*PPI*).

Nearly all the cells (>90%) in the HLA A2-PPI_15–24_ tetramer-enriched T-cell line stained with HLA A2-PPI_15–24_ tetramer when the optimized protocol containing PKI and cross-linking Ab was used. In parallel, the standard protocol that omitted these steps stained only 35% of this T-cell line, and with a low mean fluorescence intensity (Figure 4A). Over 70% of the cells in this T-cell line specifically responded to exogenously supplied PPI_15–24_ peptide indicating that roughly half of the functional T-cells could not be stained using standard pMHC tetramer staining (Figure 4B). We next subjected this enriched T-cell line to cloning by limiting dilution. Of the 34 T-cell clones generated, 33 were responsive to PPI_15–24_ peptide. All 33 peptide-responsive T-cell clones stained well with HLA A2-PPI_15–24_ tetramer when the optimized protocol was used but failed to stain without addition of PKI and cross-linking Ab. An example staining for one clone, GD.PPI.1, is shown in Figure 4C. These results provide further evidence that standard pMHC tetramer staining fails to detect fully functional PPI-specific T-cells. Peptide titrations (data not shown) with these T-cell clones indicated that they required at least 100 nM exogenous PPI_15–24_ peptide in order to activate (Figure 4D). In order to determine whether such cells might be capable of responding to endogenously processed and presented peptide antigen we used “surrogate β-cell” K562 targets expressing both HLA A2 and PPI (42). T-cell clone GD.PPI.1 did not respond to K562 cells expressing HLA A2 alone but activated in response to these cells when they were also transduced with PPI (Figure 4D). We conclude that standard pMHC tetramer staining can fail to detect fully functional diabetogenic T-cells capable of responding to endogenously expressed β-cell-specific proteins.

### Optimized pMHC Multimer Staining Is Compatible With Magnetic-Bead-Based Cell Isolation

**Figure 5:**
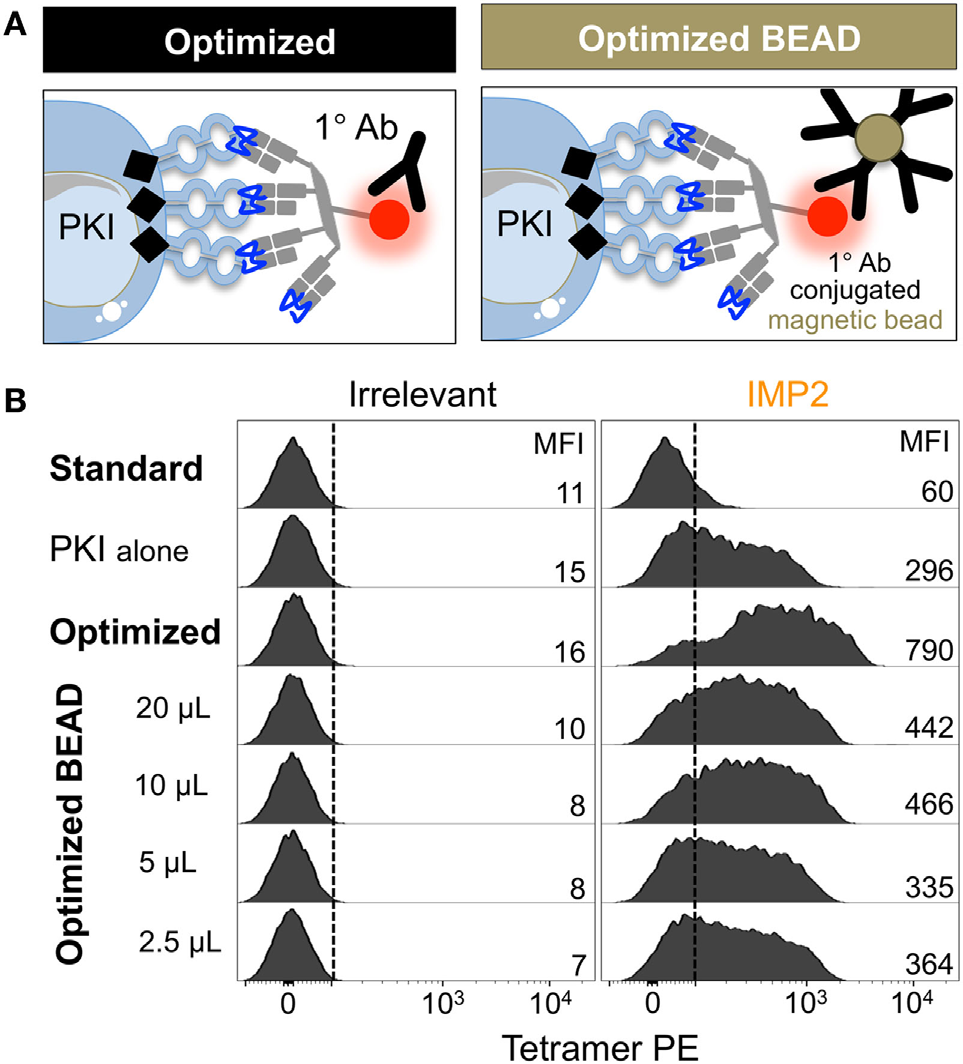
Anti-fluorochrome magnetic microbeads enhance the staining of T-cells with tetramers. **(A)** The ability of a primary (1°) unconjugated anti-fluorochrome antibody (Ab) to stabilize tetramer was also tested in the form of Ab-conjugated magnetic microbeads. **(B)** HLA A*02:01-restricted Melanoma reactive clone CR.NLS.3 was stained with irrelevant ILAKFLHWL (hTERT_540–548_) and index NLSALGIFST [Insulin-like growth factor 2 mRNA binding protein 2 (IMP2_367–376_)], PE-conjugated tetramers using the conditions shown. All samples were treated with PKI apart from the standard conditions. The anti-PE magnetic microbeads were used as recommended by the manufacturer (Miltenyi Biotech), 20 µL per 100 µL of staining volume for up to 1 × 10^7^ cells, or bead volume dilutions thereof. The MFI of staining is shown for each condition.

Flow cytometric sorting can be detrimental for T-cell fitness, and in some cases >98% of sorted cells fail to expand in culture (59). We have previously used an optimized protocol for isolating rare pMHCII-responsive CD4^+^ T-cells incorporating a magnetic bead enrichment step (60). These beads capture pMHC multimer^+^ cells *via* anti-fluorochrome Ab. We, therefore, reasoned that much of the improvement in T-cell recovery that we observed in our previous study (60) might have been due to stabilization of pMHC tetramer staining afforded by the anti-fluorochrome Ab-conjugated magnetic microbeads. We tested whether anti-PE microbeads could enhance the ability to detect self-specific T-cells using a T-cell clone that requires an optimized protocol in order to stain with cognate (HLA A2-NLSALGIFST) tetramer (protocol schematic Figure S1 in Supplementary Material). If anti-fluorochrome magnetic bead-conjugated antibodies could be used to enhance staining with pMHC tetramers, this would provide a ready means for isolating cells without subjecting them to flow cytometric based sorting (Figure 5A). As expected, self-reactive T-cell clone CR.NLS.3 failed to stain with cognate pMHC tetramer using standard conditions. Inclusion of PKI increased the staining intensity, which was further augmented by addition of anti-PE-conjugated magnetic beads (Figure 5B). The beads mediated a 7- to >11-fold enhancement, compared to a 13- to >18-fold improvement seen with soluble Ab (Figure 5B; Figure S2 in Supplementary Material), with increased bead volumes not improving the tetramer staining any further (Figure S2 in Supplementary Material). The improved effect with soluble Ab may reflect improved access and far lesser steric effects compared to bead-conjugated antibody. Overall, the protocol should allow magnetic sorting of tetramer^+^ cells without subjecting them to the rigors of flow cytometric sorting.

**Figure 6:**
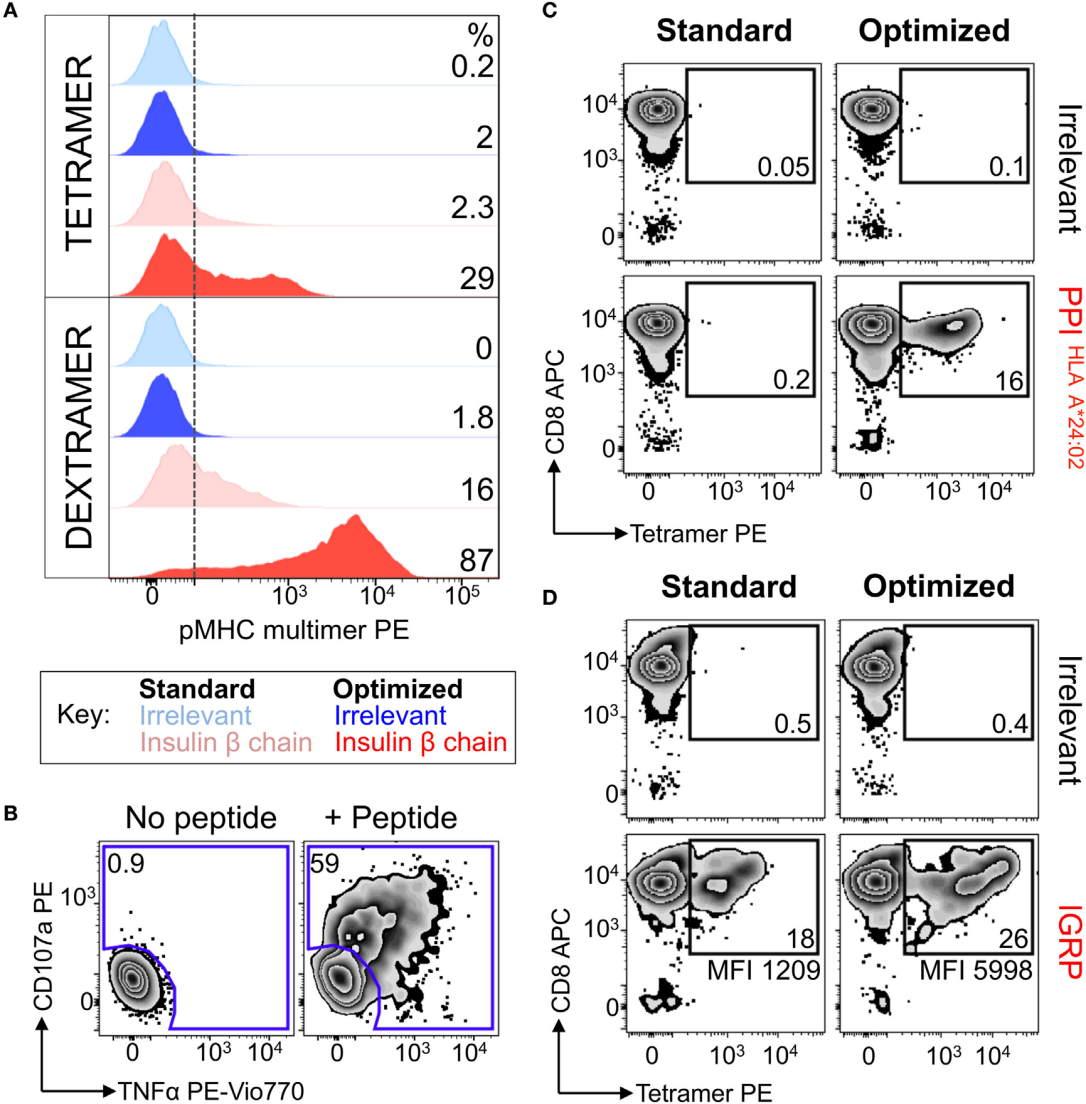
Dextramers used with magnetic based purification allowed autoimmune T-cells to be isolated directly *ex vivo*. **(A)** Peripheral blood mononuclear cells from a HLA A*02:01^+^ patient with type I diabetes were treated with PKI and then stained with insulin-β chain_10–18_ (HLVEALYLV) PE-conjugated dextramers. Post staining the cells were labeled with anti-PE antibody-conjugated microbeads and magnetically enriched. T-cells were grown *in vitro* for 3 weeks then stained with PE-conjugated insulin-β chain tetramer and dextramers, with hTERT_540–548_ (ILAKFLHWL) multimers as an irrelevant control. Staining was performed under standard (multimer alone) or optimized (PKI + anti-PE Ab) conditions. The dotted line depicts the baseline for staining based on irrelevant multimers, with the percentage of cells staining above this shown for each condition. **(B)** The magnetically enriched T-cell line generated in A was tested for reactivity against insulin-β chain peptide by incubation with TAPI-0 and staining for TNF and CD107a. The percentage of cells residing in each gate is shown. **(C,D)** CD8 T-cells from an HLA A*02:01^+^A*24:02^+^ type 1 diabetes patient were enriched using the same approach shown in A, using either HLA A*24:02-PPI_3–11_ (LWMRLLPLL) dextramer **(C)** or HLA A*02:01-IGRP_265–273_ (VLFGLGFAI) dextramer **(D)**. Two weeks post expansion the cells were stained with their respective tetramers. hTERT tetramers were used as an irrelevant control. The percent of gated cells is shown and the mean fluorescence intensity for the PPI or IGRP staining displayed.

In light of the fact that magnetic anti-fluorochrome Ab could enhance pMHC multimer staining, we next used this protocol to isolate and culture diabetogenic T-cells from the PBMC of another HLA A*0201 T1D patient. Patient PBMC were stained with insulin-β chain dextramer under optimal “bead” conditions, and purified cells were expanded using PHA and allogeneic PBMCs prior to further analyses. No staining was performed on the PBMC before sorting, as sample size was limited (5 × 10^6^ PBMCs). Limited cell number is commonplace for clinical samples due to distribution among research projects, and ethical guidelines on how much blood can be taken. The enriched line was 87% dextramer^+^ using the optimal staining protocol, with cells also staining with dextramer under standard conditions (16%) and with the optimized tetramer protocol (29%) (Figure 6A). Staining was low/absent when using standard tetramer staining and suggested that very few, if any, antigen-specific T-cells would have been isolated if standard staining had initially been used. It might have been possible to isolate cells with optimal tetramer or standard dextramer protocols but the optimal “bead” dextramer approach we applied gave a combination of superior staining and potential for magnetic enrichment. The T-cell line produced in this way showed specific reactivity for the Insulin-β chain peptide (Figure 6B) confirming that the purification process using dextramers and magnetic beads had been successful. Similarly, PPI (residues 3–11, HLA A*24:02 restricted) and IGRP (residues 265–273, HLA A*02:01 restricted)-specific T-cells were successfully enriched using dextramers and the optimal bead method for another patient. Subsequent staining of the cells with tetramers showed that PPI-specific T-cells could only be detected when the optimal protocol was used (Figure 6C). A less dramatic difference in terms of cell numbers between standard and optimized protocol was observed in case of the IGRP specific T-cells—however, the staining intensity was increased nearly fivefold in the latter case (Figure 6D). These results demonstrated that optimized pMHC multimer staining could be successfully combined with anti-fluorochrome Ab-conjugated beads to enable isolation and culturing of antigen-specific autoimmune T-cells. We next set out to apply this technique to obtain autoimmune T-cells from ankylosing spondylitis patients.

### Optimal Staining and Isolation of Antigen-Specific T-Cells From Ankylosing Spondylitis Patient Samples Using HLA B*27:05 Tetramers

**Figure 7:**
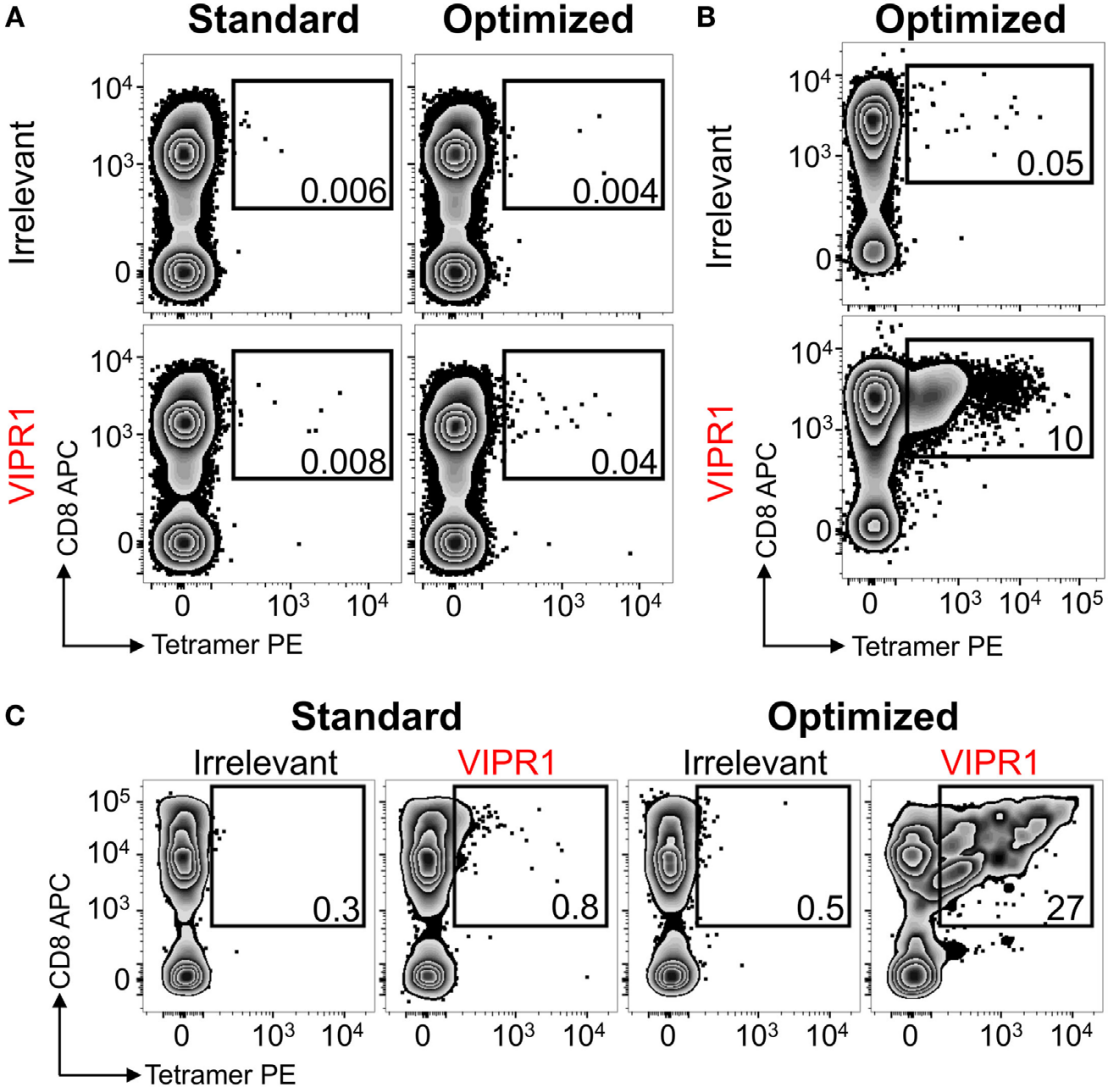
HLA B*27:05 vasoactive intestinal polypeptide receptor 1 (VIPR1) tetramers used with optimal staining conditions allowed autoimmune T-cells to be detected and isolated from patients with ankylosing spondylitis. **(A)** Peripheral blood mononuclear cells (PBMCs) from a HLA B*27:05 patient with ankylosing spondylitis were stained with irrelevant (HIV p24 gag_263–272_; KRWILLGLNK) and VIPR1_400–408_ (RRKWRRWHL) PE-conjugated tetramers directly *ex vivo* under standard (tetramer alone) and optimal (PKI + anti-PE Ab) conditions. **(B)** PBMCs for the same patient in A were PKI treated and stained with PE-conjugated VIPR1_400–408_ tetramers then labeled with anti-PE antibody-conjugated microbeads for magnetic purification. After 3 weeks of culture, the line was stained with irrelevant (p24 gag) and VIPR1 tetramer under optimal staining conditions. **(C)** Using the same approach as in B from a second patient. Cells were stained 3 weeks post purification with irrelevant (HIV p24 gag_263–272_) and VIPR1_400–408_ tetramers using standard and optimal staining protocols.

**Figure 8:**
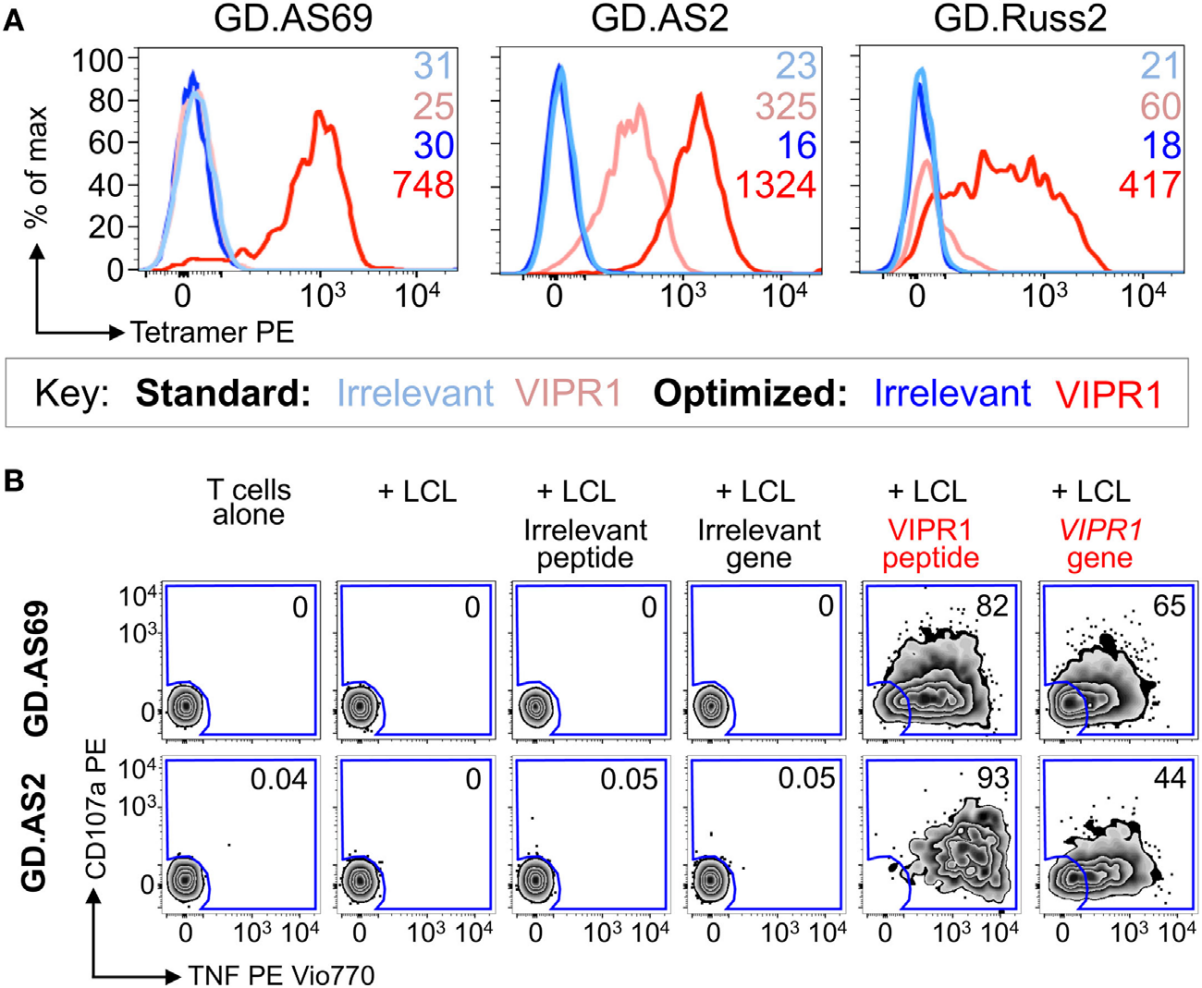
CD8 clones isolated from an ankylosing spondylitis patient using optimal tetramer staining are fully functional. **(A)** CD8 T-cell clones grown from T-cell lines (Figure 7) generated following tetramer enrichment using an optimal magnetic bead protocol (Figure 5). GD.AS69, GD.AS2, and GD.Russ2 were stained with HIV p24 gag_263–272_ (KRWILLGLNK) and VIPR1_400–408_ (RRKWRRWHL) PE-conjugated HLA B*27:05 tetramers, using standard (tetramer alone) and optimized (PKI + anti-PE Ab) protocols, according to the key. The MFI of staining is shown. **(B)** Functional testing of clones GD.AS69 and GD.AS2 using a TAPI-0 assay with CD107a and TNF Abs. Patient autologous lymphoblastoid cell line (LCL) was used to present HLA B*27:05 irrelevant peptide (DRASFIKNL from the α2 domain of collagen type VI_114–122_) and cognate VIPR1_400–408_ peptide (RRKWRRWHL). Autologous LCL were lentivirally transduced with genes for the α2 chain of collagen type VI (control protein) and vasoactive intestinal polypeptide receptor 1 (VIPR1) and used in the activation assay. Percentage reactivity is shown for the CD107a^+^ TNF*^+^* gate.

Ankylosing spondylitis (AS) is a HLA B*27:05 associated autoimmune disease with characteristic inflammation of the joints and spine (61). HLA B*27:05 tetramers refolded with the RRKWRRWHL peptide (residues 400–408 from VIPR1) were used for *ex vivo* staining of a PBMC sample from an AS patient. There was a fivefold increase in VIPR1 tetramer staining using the optimal protocol (0.04%) compared to the standard approach (0.008%), with similar irrelevant tetramer staining for both conditions (Figure 7A). Using optimal “bead” staining and magnetic separation, the VIPR1 tetramer^+^ cells were enriched to 9.7% when re-stained using the optimal protocol (Figure 7B). The same approach was used for a second AS patient (Figure 7C). Clones were obtained from both enriched lines by limiting dilution and characterized with VIPR1 tetramer. T-cell clones GD.AS69 and GD.AS2 were grown from the line shown in Figure 7B. The GD.AS69 clone needed the optimal protocol to stain with VIPR1 tetramer (Figure 8A). Conversely, clone GD.AS2 did stain under standard conditions but the optimal protocol substantially increased the intensity of staining (Figure 8A). Clone GD.Russ2, derived from the second donor, required the optimal protocol to stain with VIPR1 tetramer, similarly to GD.AS69 (Figure 8A). Clones GD.AS69 and GD.AS2 responded to exogenous VIPR peptide and were sufficiently sensitive to recognize an autologous B-cell line transduced to express the VIPR1 protein (Figure 8B). Thus, optimized pMHC multimer staining allowed successful culture and cloning of VIPR-reactive, HLA B*27:05-restricted T-cells. Two of three clones generated could not be stained with standard pMHC multimer staining protocol, and therefore, would not have been isolated without the optimized technique applied here.

## Discussion

### Standard pMHCI Tetramer Staining Fails to Detect Antigen-Specific T-Cells With Low-Affinity TCRs

Peptide–MHC multimers are widely accepted as the “gold standard” for the detection and isolation of antigen-specific T-cells. As these reagents rely on physical detection, cognate cells can be detected without relying on any particular effector function. Physical detection of T-cells has some further advantages over functional detection as cells that are in the process of dividing would be unlikely, or unable, to respond by killing a target or secreting a lymphokine, and therefore, would remain undetectable. When used optimally, pMHC multimers usually detect a larger population of antigen-specific T-cells than responding cells in a functional assay. In keeping with this observation, we consistently found more antigen-specific cells with optimal pMHC multimer staining than were detected in parallel functional assays. Accordingly, throughout our dataset, the fraction of T-cells detected with optimal pMHC multimer staining was higher than that detected in functional assays (detecting CD107a and/or TNF). Conversely, standard staining protocols detected a fraction of cells that was generally smaller than that revealed by functional assay confirming that some functional T-cells were not detected. These observations are in line with tetramer staining verses functional readouts using monoclonal T-cell populations where all cells might be expected to have capacity to respond to relatively high concentrations of exogenous cognate peptide. In parallel comparisons, 99.6% of the 1E6 T-cell clone stained with cognate tetramer while only 27 and 20% responded by intracellular staining for TNF in response to 1 µg/mL of cognate peptide or target cells expressing the cognate HLA A2 and PPI target, respectively (42). Similarly, 99% of GAD65-specific T-cell clone RK9C10 stained with pMHC tetramer while only 50.6 and 62% responded by intracellular TNF and surrogate degranulation marker CD107a staining, respectively (56). We provide three further examples of parallel tetramer staining and activation of T-cell clones in Figure S3 in Supplementary Material, while all three clones exhibit >99% tetramer staining their activation with 10 µM cognate peptide or real melanoma target cells ranges from 52 to 83%. Thus, up to half of cognate T-cells that express TCR and that can be stained with pMHC multimer fail to respond in effector function assay.

The original pMHC multimer, a streptavidin-based pMHC tetramer (18), is still the most common form in use as it can be easily manufactured in-house (as here), or acquired *via* the NIH Tetramer Core Facility (Emory) or various commercial sources. Unfortunately, the TCR-pMHC affinity threshold amenable to detection by standard pMHC tetramer staining is higher than that required for efficient T-cell activation (21). Thus, these reagents can fail to detect fully functional T-cells with low-affinity TCRs and thereby underestimate the size of antigen-specific T-cell populations. This effect can be particularly pronounced for self-reactive T-cells, or MHCII-restricted T-cells, where TCR–pMHC interactions are weaker (10). Indeed, several recent studies by different research groups have reported that, with some antigens, standard pMHC multimer staining can fail to stain the *majority* of antigen-specific T-cells (23, 33, 62). It has even been suggested that this discrepancy and the prominent use of pMHC staining for T-cell detection has biased T-cell immunology toward the type of T-cells that can be readily detected with these reagents because the important, low-affinity TCRs that can dominate antigen-specific T-cell responses are ignored (63, 64). The aim of this study was to compare the recovery of antigen-specific, self-reactive T-cells from patient samples using standard pMHC staining or an optimized protocol and to compare the results to functional assays for T-cell activation.

### An Optimized pMHC Multimer Protocol Can Recover Autoimmune T-Cells

We have developed a variety of “tricks” in an attempt to circumvent the problem of unsuccessful staining of T-cells bearing weak affinity TCRs using the standard procedures (17, 20). The simplest, and least expensive, of these improvements are the addition of PKI to prevent TCR triggering and downregulation (32) and the addition of an Ab (usually anti-fluorochrome) to cross-link the pMHC multimers (33). The advantages of these procedures are additive, and they can be applied with any pMHC multimer in all species tested to date for a cost of less than $0.05 per stain. Application of this optimized procedure in this study routinely detected an average of 40.5-fold (range 1.4- to 198-fold) more antigen-specific T-cells than parallel assays in the absence of PKI and anti-fluorochrome Ab. Whereas standard staining routinely discovered a smaller population of antigen-specific T-cells than could be detected in functional assays, the optimized protocol always detected more cells than parallel functional assays. As discussed above, the difference between the size of antigen-specific T-cell populations recovered by optimized pMHC tetramer staining and that revealed in functional assays might represent those cells that are undergoing cell division or happen to express a low level of TCR on their surface. We routinely observe that less cells respond functionally than stain with pMHC multimer even within clonal T-cells (Figure S3 in Supplementary Material). Overall, our results show that optimized protocols are substantially better at detecting self-reactive T-cells than standard protocols. The question remains whether the “optimal” protocol using pMHC dextramer + PKI + Ab recovers all the T-cells capable of responding to a given pMHC, or whether there are functional T-cells bearing TCRs too weak to be captured using even this optimized procedure.

### Are Higher Order pMHC Multimers Better?

We have been asked this question many times since we compared staining with pMHC tetramers to that with pMHC dextramers in parallel assays and found that the higher order multimers, which carry more copies of both pMHC and fluorochrome per molecule, could recover CD4 and CD8 T-cells with weaker TCRs (22). Subsequently, Davis and colleagues showed that pMHC dodecamers, which incorporate 12 pMHC per molecule, stained twofold to fivefold more murine and human CD4 and CD8 T-cells than detected in parallel assays with pMHC tetramers (23). These findings make it clear that, for T-cells with low-affinity TCRs, higher order multimers can beat pMHC tetramers in head-to-head assays using the same conditions. However, the threshold of TCR affinity amenable to detection with pMHC tetramers with the optimal protocol used here is very low and below that found on the vast majority of cognate T-cells. We also find that an optimal staining with pMHC tetramer can recover cells that cannot be detected using standard pMHC dextramer protocols as currently listed on the Immudex website (March 2018). We regularly see T-cells that can be stained with optimized pMHC tetramer staining that cannot be stained by standard pMHC dextramer staining in the absence of the PKI + Ab; this threshold is graphically depicted in Figure 1. The staining of the dextramer-sorted insulin-β line in Figure 6 provides a good example of these sensitivities; staining of this line was greatest using dextramer + PKI + Ab (87%) compared to 29, 16, and 2.3% for optimized tetramer staining, standard dextramer staining, and standard tetramer staining, respectively. Optimized pMHC tetramer staining was readily able to detect the PPI-specific 1E6 T-cell clone when it was spiked into a PBMC samples (33); this T-cell is known to bear a very weak affinity TCR [*K*_D_ > 240 μM (15, 65)]. Optimized pMHC tetramer staining can also recover the ILA1 T-cell clone from PBMC using the 5Y variant of the ILAKFLHWL hTERT-derived (41) peptide which similarly has a *K*_D_ ~ 250 μM (33). Optimized pMHC tetramer staining with the 8E variant in this system, which is estimated to bind with *K*_D_ ~ 2 mM (and >500 μM with certainty), did not recover the ILA1 T-cell in parallel experiments (33). The fact that optimized pMHC dextramer staining with the 8E variant can fully recover the ILA1 T-cell clone in parallel experiments provides some indication of where the threshold of detection for optimized pMHC tetramer and dextramer must lie. However, pMHC multimer binding thresholds are dependent on both TCR and CD8 expression levels so can vary with time for cultured cells, or if cells have recently encountered cognate antigen *in vivo*, so the above should only be taken as a rough estimate. In summary, all but T-cells bearing the very weakest of cognate TCRs can be recovered using an optimized pMHC tetramer protocol. Thus, any pMHC multimer with 4 or more pMHC per molecule should be sufficient for almost all purposes providing an optimized protocol including PKI and cross-linking Ab is adopted, and we believe that the advantages afforded by higher order multimers will only rarely be required.

### Are T-Cells With Very Low-Affinity TCRs Truly Functional?

It is becoming increasingly clear that optimized pMHC multimer staining procedures can stain T-cells with cognate TCRs that are far weaker than were previously believed to have any possibility of being functional. Consequently, it is worth asking two questions: (1) Can extremely weak TCR interactions trigger and activate T-cells? And (2) do low-affinity TCRs make a significant contribution to immunity? These important points will be addressed below.

#### Extremely Weak TCR Interactions Can Activate T-Cells

The best model system we are aware of for addressing this issue is the ILA1 T-cell/TCR, which recognizes the hTERT-derived peptide ILAKFLHWL (41). The ILA1 TCR refolds reasonably well to allow biophysical and structural studies with the soluble extracellular domain (66). As described above, the 5Y and 8E variants of ILAKFLHWL bind to the ILA1 TCR with *K*_D_s of >240 μM and >500 μM, respectively (21). The 5Y and 8E variants of ILAKFLHWL act as reasonable agonists of the ILA1 T-cell; although these ligands are completely dependent on MHC interaction with the CD8 coreceptor (67). Thus, this model system suggests that extremely weak TCR interactions can activate T-cells. In the real world, the PPI peptide-specific 1E6 TCR, identified in a patient T-cell that can kill human pancreatic β-cells (42), thus demonstrating responsiveness to natural target cells, has a TCR affinity of >240 μM (15, 65). In this study, we found that optimized pMHC tetramer staining recovered less T-cells from an insulin-β CD8 T-cell line than could be detected by functional assay (Figures 6A,B) and that standard pMHC dextramer staining only detected 35% of a PPI-specific T-cell line where >70% of T-cells responded to cognate peptide in a functional assay (Figure 4). The T-cells that responded to peptide but were not recovered using pMHC staining in these assays presumably express TCRs with extremely weak affinity for the cognate antigen. Based on the available evidence, we conclude that extremely weak TCR interactions can indeed activate T-cells but this does not prove that such T-cells are of biological relevance, particularly if they are competing for antigen *in vivo* with T-cells that have higher affinity TCRs.

#### Low-Affinity TCRs Make a Significant Contribution to Immunity

Our own data show that T-cells with low-affinity TCRs can function relatively poorly (12, 13, 21). These data fit with the general picture that has emerged from classic kinetic proofreading models of TCR triggering and T-cell activation (68, 69). Thus, there is now a general belief that T-cells that are most sensitive to antigen will bear the highest affinity TCRs and that these T-cells will, by definition, be avid binders of soluble pMHC multimers. The flipside of this viewpoint is that T-cells with weak affinity TCRs will be insensitive to peptide antigen and will be unlikely to play a dominant immune role *in vivo*. Our faith in the importance of TCR affinity and the emergence of pMHC tetramers as a gold standard for T-cell detection may have been responsible for study bias which underestimates the role of low-affinity TCRs (63). In the absence of definitive proof, assumptions that staining with pMHC multimer is a surrogate of how antigen-sensitive a given T-cell might be and of how effective and important it is *in vivo* are precarious. As discussed below, the view that T-cells with low-affinity TCRs are relatively insignificant during regular immune responses has softened over the last 8 years with murine studies showing first that low-affinity TCR interactions generate a complete immune response during the first week of infection with *Listeria monocytogenes* (70) through to recent data showing that TCRs that escape negative selection and that cannot be stained with cognate pMHC tetramer have sufficient reactivity to cause disease *in vivo* (71).

##### Evidence From Humans and Other Primates

A recent video study that provided protocols for pMHCI staining of Simian Immunodeficiency Virus-specific CD8 T-cells in Rhesus Macaques showed that addition of 50 nM Dasatinib enhanced staining from 0.1% of CD8^+^ T-cells to 0.96% with a considerable concomitant increase in the MFI (34). Here, our demonstration that patient-derived T-cells with low-affinity TCRs can respond to relevant targets expressing endogenous antigen suggests that these cells have capacity to be relevant *in vivo*. Furthermore, the 1E6 TCR isolated from a T1D patient, and capable of killing HLA A*02:01^+^ pancreatic β-cells in a glucose-dependent manner (42), has a low-affinity TCR (*K*_D_ > 240 μM). Other patient-derived TCRs that see different diabetogenic epitopes also bind with *K*_D_ > 150 μM (manuscript in preparation) in line with suggestions that very weak TCRs are a common feature of autoimmune T-cells (10) (Figure 1). In accordance, a study of HLA DR-restricted responses to a type II collagen-derived peptide in a patient with relapsing polychondritis found that patient PBMC contained T-cells that could be activated by this peptide or a plate-bound form of the pMHC but could not be detected by tetramer staining with this same pMHC; these results were confirmed using patient-derived T-cell clones and attributed to autoimmune TCR-pMHC interactions being weak (72).

We have also recently demonstrated that the dominant tumor-infiltrating lymphocyte-derived clonotypes in patient blood following successful “cure” (complete lasting remission) of Stage IV melanoma can only be detected by optimized pMHC multimer staining (59). Clones expressing these TCRs were grown in culture and shown to kill autologous tumor but were only amenable to optimized pMHC multimer staining, suggesting that they bore TCRs with low-affinity for cognate antigen. These data strongly suggest that low-affinity TCRs substantially contribute to mediating remission during successful immunotherapy. We have also made the unexpected discovery that PBMC from the majority of EBV seropositive HLA A*02:01^+^ individuals contain functional CD8 T-cells specific for the immunodominant BMLF1-derived epitope GLCTLVAML that can only be stained with the optimized procedure described here. PBMC from one individual made a strong response to GLCTLVAML peptide but showed very poor staining with pMHC tetramer in the absence of PKI and cross-linking Ab (59). Optimized staining revealed a sizeable population of T-cells (0.15% of total CD3^+^ cells) that stained with high intensity. A clonal population of BMLF1-specific cells from this individual responded to 100 pM peptide and an autologous EBV-transformed B-cell line suggesting that these cells had capacity to exhibit strong potency *in vivo* (59). Davis and colleagues found that 10 nM pMHC dodecamer could recover a large population of CMV-specific T-cells from PBMC (1.76% of total T-cells); parallel staining with 10 nM pMHC tetramer recovered a population 100 times smaller (0.017%) (23). Similarly, 10 nM dodecamer of the influenza A virus HA_306–318_ epitope HLA DR4-GGPKYVKQNTLKLAT recovered a population of cells comprising 0.92% of total T-cells compared to recovery of over fourfold less T-cells with pMHC tetramer even when used at 15 times higher concentrations (23). The authors conclude that “*traditional tetramers may significantly underestimate the actual frequency of antigen-specific T-cells in the repertoire, which might be physiologically important in maintaining and mediating the effector function and homeostasis of the adaptive immune system*.” In summary, increasing evidence suggest that human T-cells that bear weak TCRs that cannot be detected with standard pMHC tetramer staining play an important immune role. Nevertheless, it is impossible to definitively prove that these cells are important *in vivo*.

##### Evidence From Mice

Although there is abundant recent evidence demonstrating that low-affinity TCRs can make a profound contribution to immunity, the counterview suggested in older studies is still prevalent. Unfortunately, most studies of TCR affinity and T-cell activation in mice have been undertaken in transgenic animals where all T-cells express a single, high-affinity TCR. Zehn and colleagues broke this mold by deliberately constructing a mouse expressing a TCR that barely escaped from negative selection (i.e., a TCR with affinity for self-antigen at the threshold of affinity that determines whether a T-cell is culled by negative selection or survives central tolerance mechanisms) (71). The resultant low-affinity OT3 T-cells are below the limit for reliable detection by conventional tetramer staining. However, OT3 T-cells, developed into functional effector and memory T-cells, had sufficient reactivity to cause disease and could respond to ligands that bound with an affinity well below the affinity threshold for negative selection (71). These even weaker ligands would have almost certainly not stained OT3 T-cells with standard pMHC tetramer staining technology, yet they were capable of inducing acute activation in the periphery. This well-defined model of autoimmune T-cells *in vivo* demonstrated that T-cells with extremely weak TCR–pMHC interactions, well below the threshold for pMHC multimer staining, can make a profound contribution to tissue destruction. Earlier studies, by Zehn et al., in the high-affinity OT1 transgenic model first gave hints that very weak TCR ligands could be functional and showed that such interactions were sufficient to induce initial rapid expansion of naïve T-cells to generate effector and memory cells, thereby challenging the view that strong TCR–pMHC interaction were a prerequisite of CD8 T-cell activation (70). Even the V4 variant of the OT1 cognate OVA-derived SIINFEKL peptide expanded OT1 cells *in vivo* when delivered in recombinant *L. monocytogenes* (70). Remarkably, the OT1 TCR binds to the V4 variant with a *K*_D_ of >1 mM by SPR (4); yet, this ligand could induce similar proliferation to the wild-type OVA ligand over 6–7 division cycles and produce cells with a similar effector phenotype 1 week after infection despite having an affinity well below the threshold for negative selection (70). The only major difference observed between a strong TCR stimulus and an extremely weak stimulus occurred at later time points and affected the length of the expansion period and the concomitant burst size (i.e., responses induced by very weak TCR ligands were curtailed). Despite the difference in burst size, the primed T-cells developed into memory cells that were comparable after adoptive transfer and challenge with *L. monocytogenes* expressing wild-type SIINFEKL sequence indicating that recall expansion was independent of the priming antigen stimuli (70).

Despite the compelling data described above, showing that low-affinity TCR–pMHC interactions can be extremely relevant *in vivo*, these transgenic models may bear little relevance to the real-world situation where polyclonal T-cell populations compete with each other for limited antigen. Evidence that TCR affinity may set the basal activity of T-cells by regulating the expression of restricting phosphatases such as SHP-1 (73), suggests that any preference for high-affinity TCRs could be offset by counterbalancing mechanisms that “level the playing field” and allow weak affinity TCRs to compete with their more avid counterparts. Recent data from Evavold and colleagues examining six different polyclonal CD4 T-cell responses in mice challenges the notion that T-cells with high-affinity TCRs, which are amenable to detection with standard pMHC tetramer technology, are the major responders during primary immune responses (64). Instead, these data show that T-cells that stain by standard tetramer technology comprise just 5–30% of the total antigen-specific naïve murine T-cell repertoire. A study of TCR diversity and T-cell sensitivity in humanized HLA DR4 mice immunized with the human cartilage gp-39_263–275_ epitope uncovered great variability in the ability of HLA DR4-gp-39_263–275_ tetramers to stain cognate hybridomas. Over 30% of the hybridomas that responded to cognate antigen could not be tetramer stained leading the authors to suggest that “*immune responses that are dominated by relatively low-affinity TCR interactions, such as those that may occur in autoimmune disease, will be difficult to detect using standard tetramer techniques*” (74). Mass cytometry (CyTOF) experiments using 5C.C7 T-cells which bear a low-affinity TCR (75) support conclusions that pMHC tetramers fail to detect the majority of antigen-specific T-cells by showing that that a major population of tetramer-negative T-cells that only stained with higher order dodecamer, exhibited equal functionality to T-cells that stained with tetramer. Additional studies from the Evavold laboratory have demonstrated that pMHCII tetramer staining underestimated the H2-IA^b^-restricted CD4^+^ T-cell population specific for lymphocytic choriomeningitis virus glycoprotein_61–80_ by fourfold and the H2-IA^b^-restricted population specific for myelin oligodendrocyte glycoprotein_35–55_ by eightfold (62). In summary, the most recent *in vivo* studies lend full support to the notion that completely functional and important T-cell responses can remain “below the radar” when it comes to detection by the “gold standard” of pMHC tetramer staining.

### Optimized pMHC Multimer Staining Is Compatible With Bead-Based Magnetic Sorting

The generation of autoimmune T-cells lines for this study greatly benefited from our discovery that anti-PE Ab-conjugated to magnetic beads improved pMHC multimer staining in much the same way as we have demonstrated for soluble anti-fluorochrome antibodies (33). This fortuitous effect then allowed magnetic sorting and culturing of T-cells that had TCRs that were below the threshold for staining by standard pMHC tetramer technology. Curiously, we previously used a technique involving such a magnetic bead step for the detection of antiviral CD4 T-cells (60). This technique appears to have become widely adopted for the characterization of CD4 T cells by pMHCII tetramers although most studies have not shown staining with and without the inclusion of beads, meaning that it is not possible to compare the intensity of staining generated with and without bead inclusion. Nevertheless, early studies using magnetic bead enrichment did show staining of samples before and after bead enrichment. Revisitation of these data in light of our discovery that anti-fluorochrome antibody-conjugated magnetic beads can stabilize pMHC multimer staining is revealing. A study of CD4 T-cells in nonobese diabetic mice used two different pMHCII tetramers to stain samples from peptide immunized mice before and after bead enrichment (76). Throughout this study, it is evident that the cells recovered with these reagents after enrichment with anti-PE magnetic beads exhibited a higher MFI than parallel samples without the enrichment (76). Magnetic bead enrichment was also used for the analysis of CD4 T-cells specific for the Hepatitis C virus directly *ex vivo* in patient PBMC (77). The inclusion of the magnetic bead step substantially aided the recovery of T-cells. Reanalysis of these data also shows that cells that underwent the procedure with anti-PE magnetic beads stained with higher MFI than staining without these beads (77). Presumably, the beads acted to stabilize the pMHC during final washing steps and flow cytometry as we have demonstrated for soluble anti-PE Ab (33). Our discovery in this study that anti-PE magnetic beads improve the staining of T-cells with low-affinity TCRs now suggests that part of the improved staining researchers observed over a decade ago was due to the effect we describe here. Indeed, had the anti-PE beads been added earlier in the protocol, it is likely that the enhancement effect would have been even more pronounced. The inclusion of anti-fluorochrome magnetic beads also improved the MFI of staining in a further study from the Klenerman laboratory that used HLA-DR-HA_306–318_ to stain PBMC directly *ex vivo* (78). Anti-fluorochrome magnetic beads have also had a positive effect on the recovery of CD8 T-cells with pMHC tetramers (79). Indeed, close scrutiny of these data from Barnes et al. (79) show that inclusion of the beads enhanced the MFI of pMHC tetramer staining and cleaned up the “tail” of tetramer^low^ cells to give clean populations of CD8 T-cells specific for EBV, parvovirus, and CMV. Although there is improvement of pMHC multimer staining with cross-linking Ab beads regardless of the TCR affinity, this effect is likely to be greatest when the TCR affinity is low and the TCR-pMHC dwell time is short (33). The ability to stain, magnetically sort, and successfully culture T-cells with low-affinity TCRs as we have done here should aid future study of such cells.

### Summary

Mounting evidence now challenges the view that only T-cells with relatively high-affinity TCRs play a prominent role in immunity. This is especially true for self-antigens where T-cells with the strongest TCRs are eliminated by central and peripheral tolerance mechanisms. Anticancer and autoimmune TCRs can bind to their cognate pMHC with very low affinities that often fall below the threshold compliant with standard pMHC multimer staining. The inexpensive and easily applied optimization methodology we describe here enables recovery of fully functional, self-reactive T-cells with extremely low TCR affinities. It remains to be seen whether the lower TCR-pMHC affinity threshold that can be detected by the optimized pMHC multimer technology is sufficient to encapsulate all T-cells that are capable of mounting a functional response to antigen. Nevertheless, we strongly recommend that researchers apply these techniques, and where possible also assay T-cell function, to avoid underestimating the size of antigen-specific T-cell populations and the diversity of antigen-specific TCRs.

## Ethics Statement

Cryopreserved PBMCs from patients with T1D were obtained with the approval of a national research ethics committee and informed consent was obtained from all participants. Ankylosing spondylitis patients were recruited locally from clinic (Cardiff Regional Experimental Arthritis Treatment and Evaluation Centre) and anonymized whole blood used for preparation of PBMCs. Patients gave informed consent in accordance with Research Ethics Committee for Wales (12/WA/0045). PBMC were obtained from a further HLA B*2705 ankylosing spondylitis patient diagnosed at the Institute of Rheumatology, Russian Academy of Medical Sciences, Moscow, Russia, in accordance with modified New York criteria. This patient gave written informed consent prior to enrolling in the study. The study was approved by the local ethical committee of Pirogov Russian National Research Medical University, Moscow, Russia.

## Author Contributions

GD, EZ, CR, AW, LY, ML, SW, and MA performed experiments, analyzed data, and critiqued the manuscript. DC, MP, and EC provided samples and patient data and critiqued the manuscript. GD and AS conceived and directed the project and wrote the manuscript. All authors contributed to manuscript revision, read, and approved the submitted version.

## Conflict of Interest Statement

AKS is an inventor of patent WO 2010032022 “Use of protein kinase inhibitor to detect immune cells, such as T-cells”. The authors declare no other conflicts of interest.
