# Supplementary material for: Optimized Peptide–MHC Multimer Protocols for Detection and Isolation of Autoimmune T-Cells
Source: Front Immunol. 2018 Jun 29;9:1378. doi: 10.3389/fimmu.2018.01378 (PMC6034003; doi:10.3389/fimmu.2018.01378)
Supplement: Supplementary file 1 [file image_1.PDF]

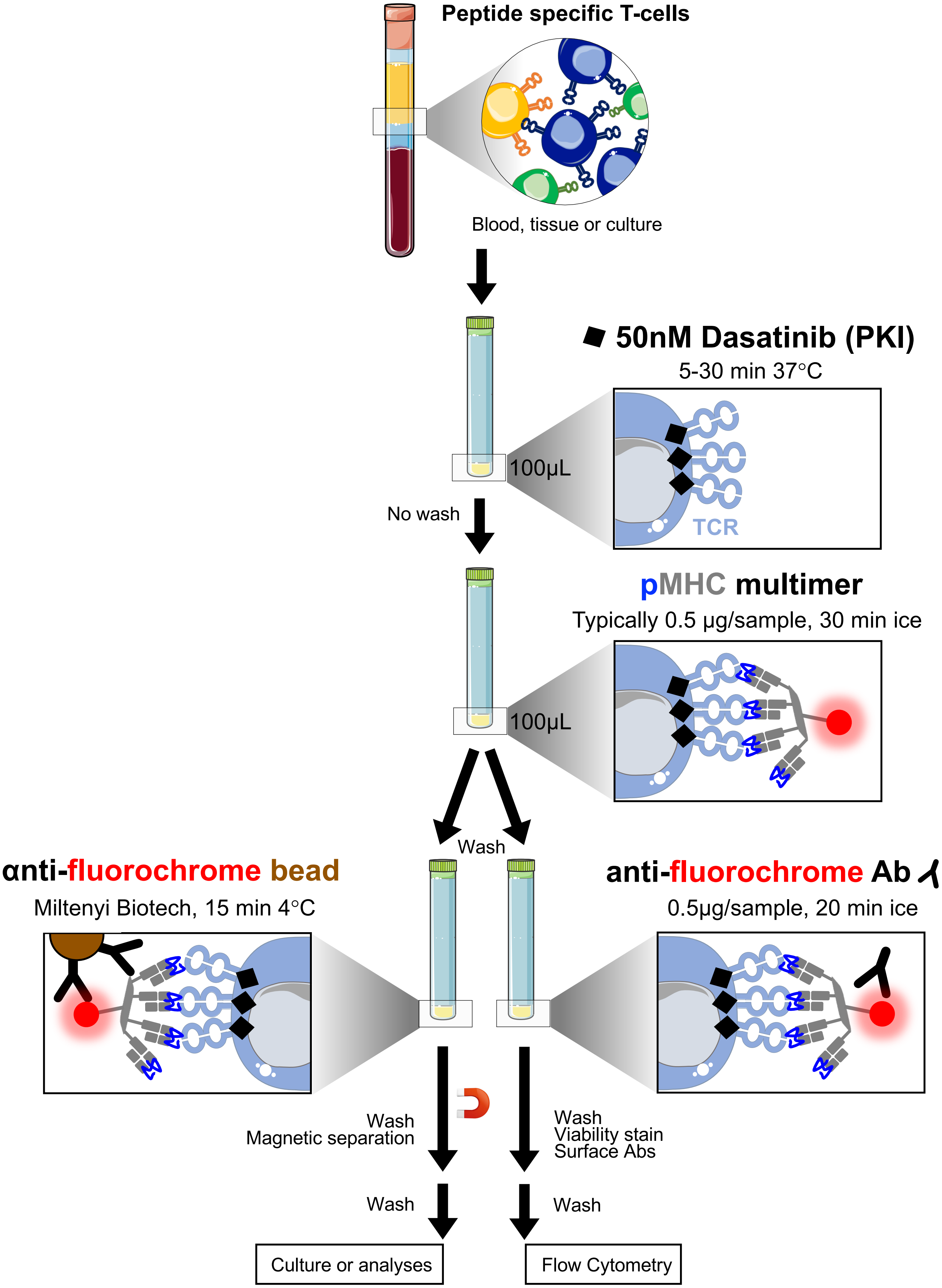

Supplementary Figure 1: Overview of the optimized staining protocols.

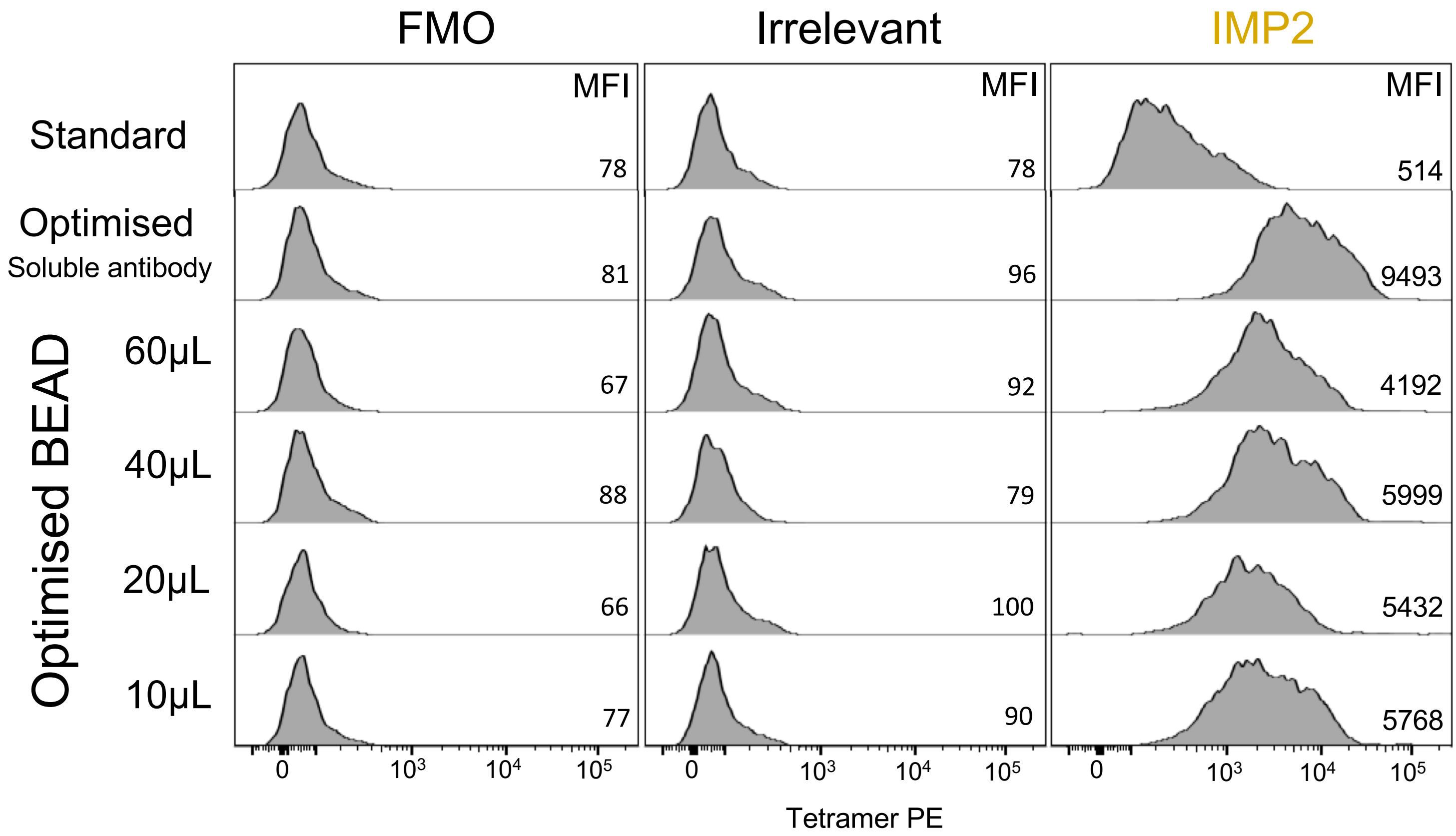

Supplementary Figure 2: Testing different volumes of anti-PE magnetic microbeads for enhanced tetramer staining. As shown in figure 5, anti-PE microbeads enhanced tetramer staining when compared to a standard staining protocol, but not to the levels seen for the soluble anti-PE antibody (optimised protocol). In light of this we increased the volume of beads to establish if the the MFI could be increased to levels seen with the soluble antibody. The HLA A*02:01 restricted melanoma reactive clone CR.NLS.5 was stained with irrelevant ILAKFLHWL (hTERT$_{540-548}$) and index NLSALGIFST (Insulin-like growth factor 2 mRNA binding protein 2 (IMP2$_{367-376}$), PE-conjugated tetramers using the conditions shown. All samples were treated with PKI apart from the standard conditions. The anti-PE magnetic microbeads were used as recommended by the manufacturer (Miltenyi Biotech), 20 μL per 100 μL of staining volume for up to 1x10^7 cells, or the bead volumes shown. The MFI of staining is shown for each condition. The reduced effect observed with bead conjugated anti-PE antibody compared to soluble antibody likely reflects differences in accessibility and steric hinderance affects.

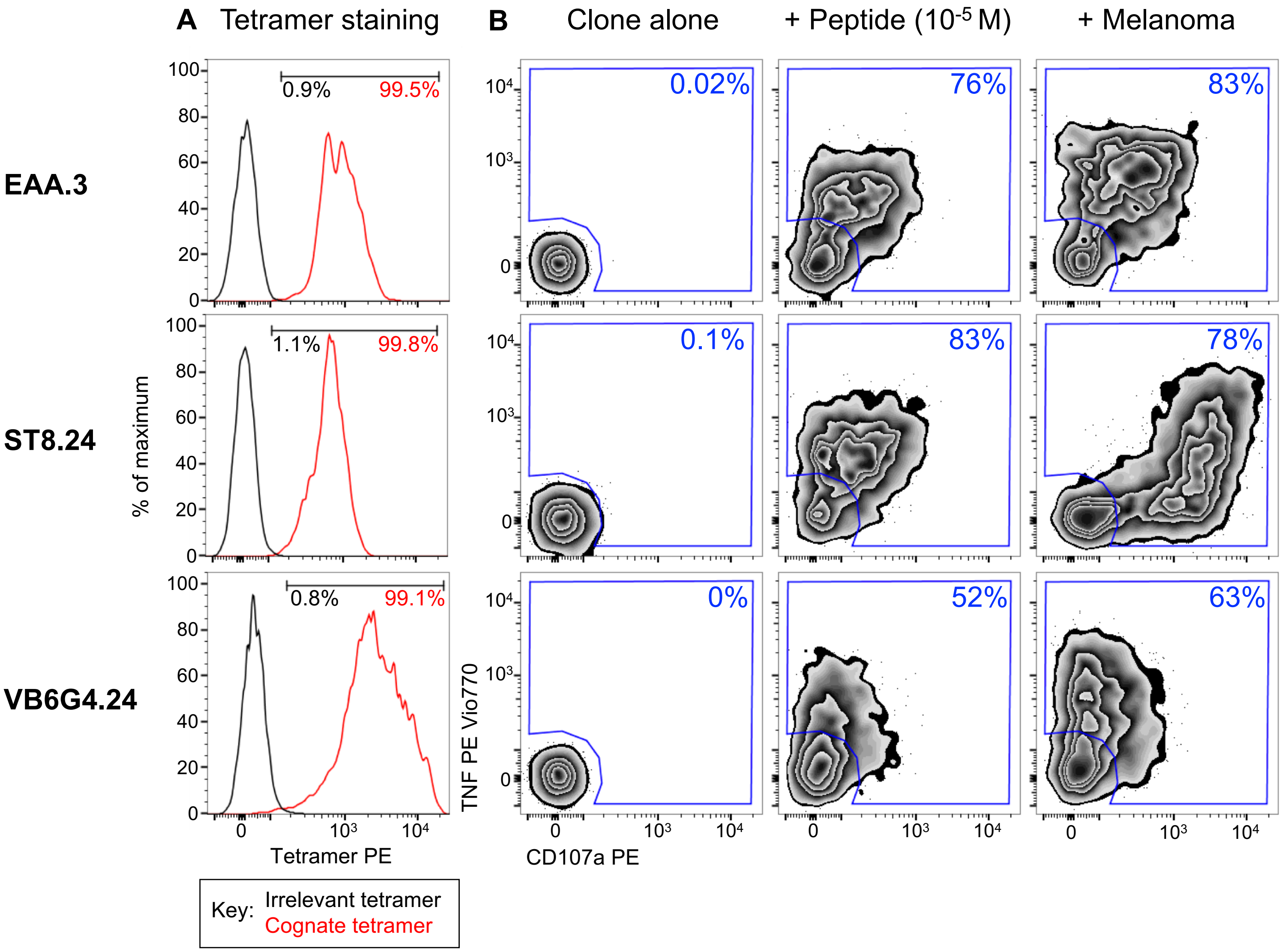

Supplementary Figure 3: Comparative tetramer and functional analyses of anti-cancer clonal T-cells. Metastatic melanoma patient derived T-cell clones EAA.3, ST8.24 and VB6G4.24 were stained with cognate tetramer under optimal conditions (PKI + tetramer-PE + primary anti-PE unconjugated antibody) (**A**) and used in activation assays (TAPI-0, with anti-CD107a and anti-TNF antibodies) for 5 h with exogenous peptide or autologous melanoma (**B**). (**A**) EAA.3 and ST8.24 where stained with EAAGIGILTV tetramer, and VB6G4.24 with E**L**AGIGILTV (heteroclitic amino acid in bold) tetramer. Irrelevant ALWGPDPAAA preproinsulin tetramers were used. The percentage of cells residing under the gate for the irrelevant (black) and cognate (red) tetramers are shown. (**B**) The peptide used for activation was the same as used for tetramer staining. The percentage of cells that resided in the CD107a+/TNF+ gate is shown.
